# *BRCA1* intronic *Alu* elements drive gene rearrangements and PARP inhibitor resistance

**DOI:** 10.1038/s41467-019-13530-6

**Published:** 2019-12-11

**Authors:** Yifan Wang, Andrea J. Bernhardy, Joseph Nacson, John J. Krais, Yin-Fei Tan, Emmanuelle Nicolas, Marc R. Radke, Elizabeth Handorf, Alba Llop-Guevara, Judith Balmaña, Elizabeth M. Swisher, Violeta Serra, Suraj Peri, Neil Johnson

**Affiliations:** 1 Molecular Therapeutics Program, Fox Chase Cancer Center, Philadelphia, PA 19111 USA; 2 Lewis Katz School of Medicine, Temple University, Philadelphia, PA 19111 USA; 3 Genomics Facility, Fox Chase Cancer Center, Philadelphia, PA 19111 USA; 4 Department of Obstetrics and Gynecology, University of Washington, Seattle, WA USA; 5 Bioinformatics and Statistics, Fox Chase Cancer Center, Philadelphia, PA 19111 USA; 6 Experimental Therapeutics Group, Vall d’Hebron Institute of Oncology, Barcelona, Spain; 7 Hereditary Cancer Genetics Group, Vall d’Hebron Institute of Oncology, Barcelona, Spain

**Keywords:** Cancer therapeutic resistance, Ovarian cancer

## Abstract

*BRCA1* mutant carcinomas are sensitive to PARP inhibitor (PARPi) therapy; however, resistance arises. BRCA1 BRCT domain mutant proteins do not fold correctly and are subject to proteasomal degradation, resulting in PARPi sensitivity. In this study, we show that cell lines and patient-derived tumors, with highly disruptive BRCT domain mutations, have readily detectable BRCA1 protein expression, and are able to proliferate in the presence of PARPi. Peptide analyses reveal that chemo-resistant cancers contain residues encoded by *BRCA1* intron 15. Mechanistically, cancers with BRCT domain mutations harbor *BRCA1* gene breakpoints within or adjacent to *Alu* elements in intron 15; producing partial gene duplications, inversions and translocations, and terminating transcription prior to the mutation-containing BRCT domain. BRCA1 BRCT domain-deficient protein isoforms avoid mutation-induced proteasomal degradation, support homology-dependent DNA repair, and promote PARPi resistance. Taken together, *Alu*-mediated *BRCA1* gene rearrangements are responsible for generating hypomorphic proteins, and may represent a biomarker of PARPi resistance.

## Abstract (web summary)

BRCA1 mutations located within the BRCT domain result in proteasomal degradation and sensitivity to PARP inhibitors (PARPi). Here, the authors report genetic rearrangements in the BRCA1 gene that generate a BRCT-less BRCA1 protein isoform, which avoids degradation and leads to PARPi resistance.

## Introduction

B*RCA1* mutations predispose carriers to an increased lifetime risk of developing cancer^1^. A recent study reported that patients harboring *BRCA1* germline mutations had a 72% and 44% cumulative risk of developing breast and ovarian cancer before 80 years of age, respectively^2^. *BRCA1* mutations are also associated with improved therapy response and survival outcomes^3,4^. Breast and ovarian cancer patients with tumors harboring somatic or germline *BRCA1* mutations have demonstrated robust and lasting responses to PARP inhibitor (PARPi) treatments^5–9^. PARPi’s selectively induce cell death in homologous recombination (HR)-deficient *BRCA1/2* mutant cells, while leaving wild-type cells that are HR-proficient intact^10,11^. Despite the overall efficacy of PARPi therapy, subsets of *BRCA1* mutant tumors have innate resistance, and others acquire PARPi resistance during the course of treatment^12^.

The *BRCA1* gene is located on chromosome 17 and consists of 24 exons, 22 of which are protein coding. Mutations associated with cancer-predisposition are found throughout the gene in all coding exons as well as exon−intron splice sites^13,14^. The longest *BRCA1* isoform generates an 1863 amino acid (aa) length protein that consists of several highly conserved domains. The N-terminal RING domain facilities heterodimerization with BARD1. Toward the C-terminal end, the coiled-coil (CC) domain interacts with PALB2 and spans approximately aa’s 1393−1424. Further downstream, the BRCT domain consists of two repeats, the first BRCT repeat includes aa’s 1642−1736 and the second repeat aa’s 1756−1855^15,16^. The BRCT domain binds to proteins containing a phosphorylated serine-proline-x-phenylalanine (pSPxF) motif, including CtIP, Abraxas, and BRIP1^17,18^.

BRCA1 plays a critical role in HR DNA repair, and mutations that disrupt protein activity result in defective HR^19–22^. Moreover, cells that are HR-deficient are highly sensitive to PARPi and platinum treatments^10,11^. BRCA1 contributes to HR at distinct steps through the formation of various protein complexes. The BRCA1−CtIP interaction has been associated with efficient DNA end resection^23–25^, and BRCA1−PALB2 interaction is required for the formation of a larger BRCA1-PALB2-BRCA2-RAD51 complex that promotes RAD51 loading^26–28^. BRCA1-BARD1 has been shown to displace 53BP1 and activate end resection^29,30^, as well as stimulate RAD51-mediated DNA joint formation^31^.

The BRCA1 BRCT domain is critical for tumor suppression, and a significant portion of germline mutations can be found in this region^32,33^. The two BRCT repeats pack together through a conserved triple-helical interface that mediate BRCT-BRCT contacts^34–36^. Previous studies using proteolysis-based assays showed that the majority of commonly arising truncating frameshift and missense mutations that occur within the BRCT domain coding sequence alter the protein folding state. In turn, unfolded and destabilized proteins were subject to proteasomal degradation^32,34,35^. However, when the entire BRCT domain is absent due to stop codons arising prior to the BRCT domain coding sequence, protein products avoid proteasomal degradation and can be abundantly expressed^37,38^. BRCA1 BRCT domain mutant cancers have previously been characterized and demonstrated low or undetectable protein expression^21,39,40^. In the current study, we identified a mechanism for generating BRCA1 isoforms that lack the entire BRCT domain (BRCTless) involving *Alu*-mediated genomic rearrangements. We show that BRCA1 BRCTless isoforms are hypomorphic and promote PARPi resistance.

## Results

### *BRCA1* intron 15 is translated in SNU-251 cells

**Fig. 1:**
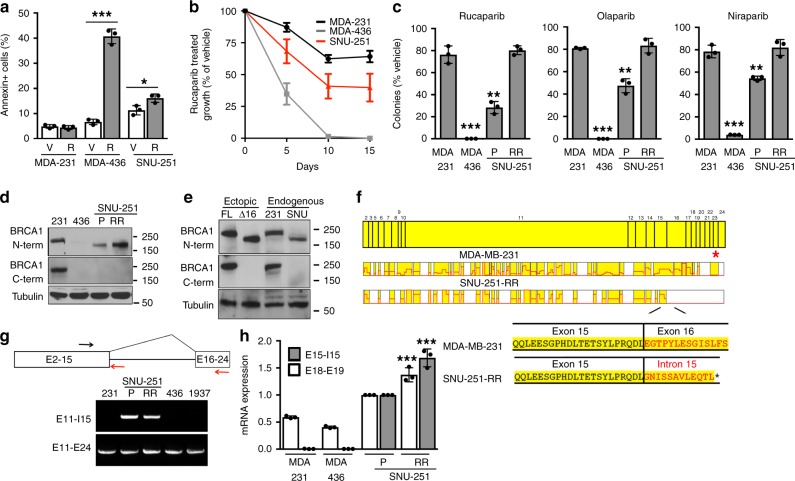
Characterization of BRCA1 in SNU-251 cells. **a** MDA-MB-231, MDA-MB-436 and SNU-251 cells were cultured in the presence of vehicle (V) or 1 µM rucaparib (R) for 5 days, followed by Annexin staining and flow cytometry. ***p < 0.001, *p < 0.05 compared to vehicle. **b** Cells were maintained in the presence of vehicle or 1 µM rucaparib and counted every 5 days. Cell growth is expressed as a percentage of vehicle-treated cells. **c** MDA-MB-231, MDA-MB-436, SNU-251 parental (P), SNU-251-RR cells were seeded at decreasing densities in the presence of either vehicle, 100 nM rucaparib, 100 nM olaparib, or 100 nM niraparib and colonies counted 2-week post-seeding. Cell survival is expressed as a percentage of vehicle-treated cells. ***p < 0.001, **p < 0.01, compared to MDA-MB-231. See Supplementary Fig. 1b for representative plates as well as γ-irradiation (IR) and cisplatin treatments. **d** MDA-MB-231 (231), MDA-MB-436 (436), SNU-251 parental (P), SNU-251-RR (RR) cells were examined for BRCA1 protein expression using N- or C-terminal-specific antibodies by Western blot. **e** MDA-MB-231 (231), SNU-251-RR (SNU) cells were assessed for BRCA1 expression using N- or C-terminal-specific antibodies by Western blot. Ectopic full-length BRCA1 (FL) and BRCA1-Δexons-16–24 (Δ16) cDNA expressing MDA-MB-436 cells were used as comparators for BRCA1 isoform molecular weights. **f** BRCA1 was immunoprecipitated from MDA-MB-231 and SNU-251-RR cells and subject to mass spectrometry. BRCA1 exons are shown and aligned with peptides that were detected (yellow). Peptides encoded by exon 15 and intron 15 were detected in SNU-251-RR cells and are shown below. **g** MDA-MB-231 (231), SNU-251 parental (P), SNU-251-RR (RR), MDA-MB-436 (436), HCC1937 (1937) cells were assessed for *BRCA1* mRNA expression by RT-PCR with the indicated forward and reverse primer locations. **h** *BRCA1* intron 15 and exons 18–19 specific mRNA expression was examined by quantitative (q)RT-PCR. ***p < 0.001 compared to SNU-251 parental cells. Data are the mean ± standard deviation (SD) of *n* = 3 biological replicates. Statistical significance was assessed by unpaired, two-tailed *t* tests.

To search for mechanisms of PARPi resistance in cancers specifically with BRCT domain-disrupting mutations, we initially characterized the effects of the PARPi rucaparib on SNU-251 cells, an endometrioid ovarian cancer cell line that carries a *BRCA1* 5564 G > A (c.5445 G > A) non-sense mutation in exon 23 ^41^. This mutation stops translation toward the middle of the second BRCT repeat after aa 1814. *BRCA1* wild-type MDA-MB-231 cells and *BRCA1* BRCT domain mutant MDA-MB-436 cells were used as comparators (Supplementary Table 1). Rucaparib treatment resulted in a small increase in annexin-positive SNU-251 cells, but markedly increased annexin-positive MDA-MB-436 cells, despite both cell lines harboring *BRCA1* BRCT domain-disrupting mutations (Fig. 1a)^39–41^. In long-term culture, while rucaparib killed MDA-MB-436 cells; SNU-251 cells demonstrated slower but maintained growth compared to vehicle-treated cells (Fig. 1b and Supplementary Fig. 1a). SNU-251 cells that were subject to long-term rucaparib culture were used in subsequent experiments, referred to as SNU-251-rucaparib resistant (RR) cells. In colony assays, MDA-MB-436 cells formed significantly fewer colonies compared with *BRCA1* wild-type MDA-MB-231 cells in the presence of the PARPi’s rucaparib, olaparib, niraparib, as well as cisplatin and γ-irradiation (IR) treatments. However, SNU-251 cells had mild reductions in colony formation, and SNU-251-RR cells formed similar numbers of colonies as MDA-MB-231 cells (Fig. 1c and Supplementary Fig. 1b).

Previously established PARPi resistance mechanisms, including reversion mutations, 53BP1 and PARP1 loss of expression, as well as BRCA1 protein folding^12,38,40,42–45^, were not detected (Supplementary Fig. 1c−g and Supplementary Data 2). Protein analyses showed BRCA1 expression was undetectable in MDA-MB-436 cells; however, SNU-251 parental cells expressed a truncated BRCA1 protein that had elevated expression in SNU-251-RR cells, and was detected with an N-terminal-specific, but not a C-terminal-targeting BRCA1 antibody (Fig. 1d). Despite lacking only 49 aa’s (6 kDa) from full-length BRCA1, the protein expressed in SNU-251 cells showed a more substantial difference in gel migration than predicted by the mutation location (Fig. 1d), and migrated in line with an ectopic *BRCA1* cDNA lacking regions corresponding to exons 16–24 (Fig. 1e). To reveal the protein identity, BRCA1 was immunoprecipitated from MDA-MB-231 and SNU-251-RR cells and analyzed by mass spectrometry. Peptide coverage was 70% and 50% in exons 2–15 coding regions, but 63% and 0% in exons 16–24 coding regions, in MDA-MB-231 control and SNU-251-RR cells, respectively (Fig. 1f). Surprisingly, we identified 12 aa’s followed by a stop codon that are predicted from translation of intron 15 mRNA (Fig. 1f). We confirmed that intron 15 was being retained in *BRCA1* transcripts in SNU-251 cells (Fig. 1g), and expression was increased in SNU-251-RR cells (Fig. 1h). The BRCA1-intron 15 containing protein (BRCA1-I15) was also the single detectable protein isoform in SNU-251 cells (Supplementary Fig. 1e), and has similar post-translational features as full-length BRCA1 (Supplementary Fig. 1f−g).

### *BRCA1* gene rearrangements generate BRCA1-I15

**Fig. 2:**
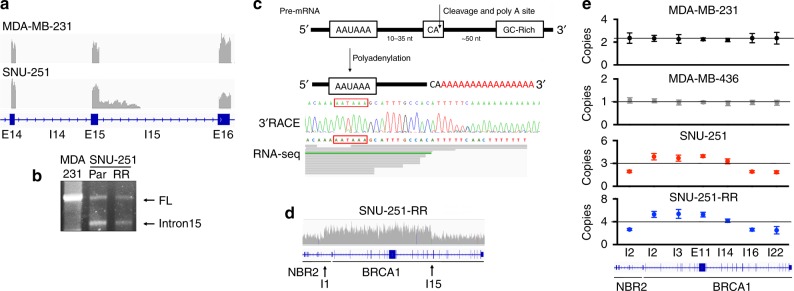
Intronic polyadenylation occurs in SNU-251 cells. **a** Integrative Genomics Viewer (IGV) showing *BRCA1* exons 14–16 reads detected using RNA-seq in MDA-MB-231 and SNU-251-RR cells. **b** Identification of *BRCA1* transcripts by 3′ RACE. Representative gel image indicating *BRCA1* full-length (FL) and intron 15 transcripts. **c** Cartoon showing a consensus AAUAAA polyadenylation signal that is followed by a CA cleavage and polyadenylation site 10–35 nucleotides downstream. Below, Sanger sequencing of *BRCA1* intron 15 mRNA identified by 3′RACE, polyadenylation sequence is highlighted. The corresponding 3′ reads detected by RNA-seq are shown for comparison. **d** IGV of SNU-251-RR WGS reads detected mapping to chromosome 17 *NBR2* and *BRCA1* gene locations. **e** Cells were assessed for genomic copy number using q-PCR at *NBR2* intron 2, *BRCA1* intron 2, intron 3, exon 11, intron 14, intron 16 and intron 22. Copy number is normalized to the signal detected in RPE nontransformed cells (*n* = 2 copy number). Line shows the mean copy number for all reactions in each cell line. Data are the mean ± SD of *n* = 3 biological replicates.

We next aimed to determine the biological mechanism of *BRCA1* intron 15 translation. DNA sequence alterations were not detected at the *BRCA1* exon-intron 15 junction (Supplementary Fig. 1h). Interestingly, in RNA-seq analyses, SNU-251-RR cells showed reads mapping to the first half of intron 15, but there was an absence of reads at the 3′ end of the intron (Fig. 2a). We also readily detected reads mapping to all exons downstream of intron 15, potentially indicating the presence of two transcripts that were terminating at the bonafide 3′UTR, and a 3′UTR located in intron 15. Indeed, two *BRCA1* transcripts were cloned and identified as full-length and intron 15-containing isoforms (Fig. 2b). Sanger sequencing of the 3′ends revealed that the intron 15 ending transcript contained a polyA tail that was approximately 18 base pair (bp) downstream of an AAUAAA polyadenylation signal sequence (chr17:43073282) and 6 bp from a CA cleavage site (Fig. 2c)^46^. Furthermore, the cloned sequence corresponded with the region of intron 15 where reads declined in RNA-seq analyses, confirming transcription termination due to intronic polyadenylation (IPA) (Fig. 2c). In whole-genome sequencing (WGS), SNU-251-RR cells showed evidence of a partial *BRCA1* copy number gain, initiating in *NBR2* intron 1, and ending midway through *BRCA1* intron 15 (Fig. 2d). A PCR-based gene copy number assay showed the partial *BRCA1* gene duplication was present in both SNU-251 and SNU-251-RR cells, with the latter gaining an additional copy of the entire locus (Fig. 2e).

**Fig. 3:**
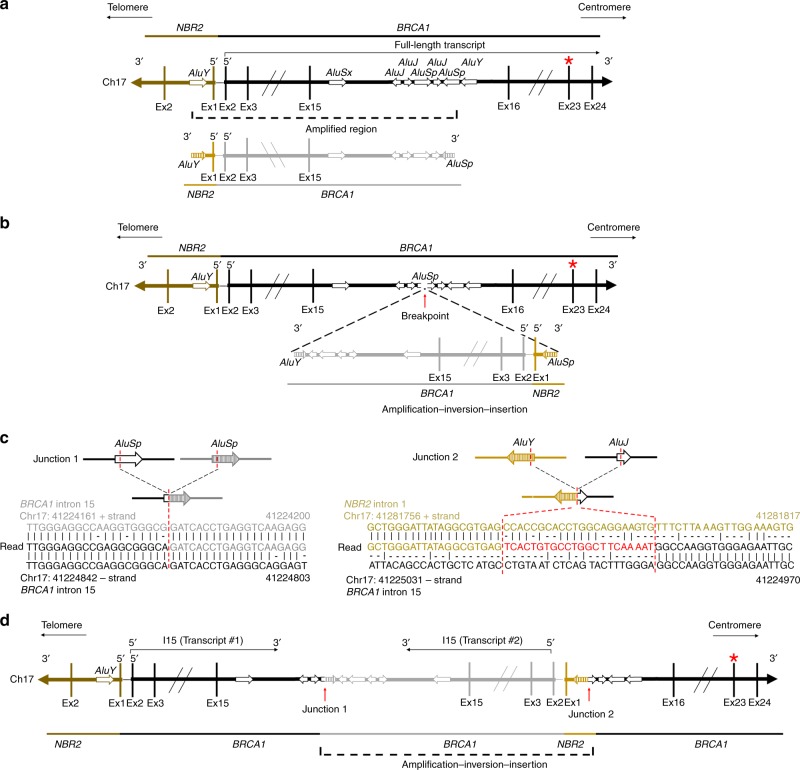
*Alu*-mediated rearrangements generate BRCA1-I15. **a** Schematic showing the *NBR2* (brown) and *BRCA1* (black) gene region of chromosome 17. *Alu* sequences are arrows and their orientation relative to the centromere and telomere of chromosome 17 are indicated. Below, the amplified region detected in SNU-251-RR cells is shown corresponding to *NBR2* (gold) and *BRCA1* (gray) sequences. The *AluY* and *AluSP* sequences at each end of the amplified region are shaded arrows. SNU-251-RR cells also harbor a non-sense mutation c.5445 G > A located in exon 23 that is indicated with a red asterisk. See Supplementary Fig. 2 and Supplementary Note 1 for more details. **b** The breakpoint and insertion site are within an *AluSp* in *BRCA1* intron 15, highlighted by a red arrow. The amplified region shown below is inverted and inserted at the indicated breakpoint. **c** *Alu* sequences that combine to form junctions 1 and 2 are shown as arrows. Reads detected by WGS that map to the indicated regions of *NBR2* intron 1 and *BRCA1* intron 15 are shown. Sequence numbers are the genomic coordinates according to the UCSC genome browser (GRCh37/hg19 assembly). Red dashed lines indicate the exact break location and the corresponding DNA sequences. The read sequence colors correspond to the *Alu* arrows above from which they derive. There is a 22 bp insertion in junction 2, indicated by red colored sequence. **d** Schematic showing the rearranged locus detected in SNU-251-RR cells. Because a polyadenylation signal exists in *BRCA1* intron 15, transcription ends at intron 15. Therefore, the amplified copy is capable of producing a second identical *BRCA1-I15* transcript. Moreover, the mutation located in exon 23 (red asterisk) is not transcribed in the rearranged locus.

Chromosome 17 is rich in short interspersed nuclear elements (SINE), specifically *Alu* elements^47–51^. A closer inspection of reads revealed that the copy number increase occurred between *AluY and AluSP* elements located in *NBR2* intron 1 and *BRCA1* intron 15, respectively (Fig. 3a and Supplementary Fig. 2)^52–54^. Further deconvolution showed that the amplified region is flipped and inserted within an adjacent *AluSP* site in *BRCA1* intron 15, chromosome 17 (Fig. 3b). Junction 1 generated a chimeric *Alu* via the joining of two homologous *AluSp* sequences, both from *BRCA1* intron 15. Junction 2 was formed by joining *AluY* and *AluJ* sequences from *NBR2* intron 1 and *BRCA1* intron 15, respectively. The latter sequences were interspaced by a 22 bp insertion that has 55% homology with the *NBR2 AluY* sequence (Fig. 3c). Of note, there was also a 167 bp duplicated sequence present at both junctions (Supplementary Note 1). Non-allelic homologous recombination (NAHR) occurs between two DNA sequences that are not alleles but share sequence similarity^48,50,55^. Given the sequence homology at junctions, we propose that NAHR may have contributed to generating this rearrangement. The final locus can produce two *BRCA1-I15* mRNA transcripts, both of which result in identical IPA events; and importantly, abrogate transcription of the mutation-containing BRCT coding region (Fig. 3d).

### BRCA1–15 promotes PARPi resistance

**Fig. 4:**
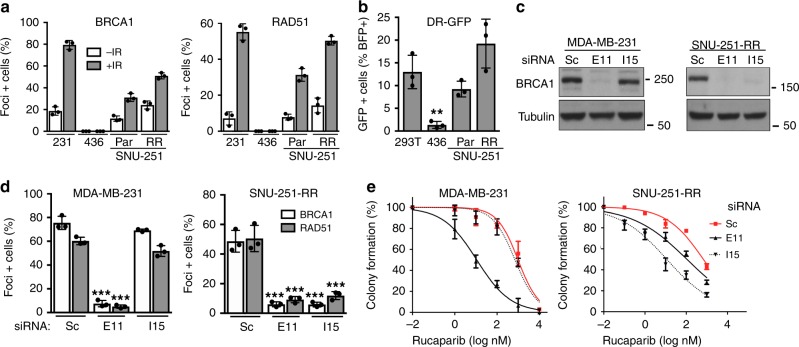
BRCA1-I15 promotes PARPi resistance. **a** BRCA1 and RAD51 foci formation was assessed by immunofluorescence (IF) with or without 10 Gy IR. See Supplementary Fig. 3b for representative images as well as γ-H2AX and 53BP1 controls. **b** HR repair capacity assessed by DR-GFP assay. **p < 0.01 compared to 293T cells. See Supplementary Fig. 3c−d for representative histograms and summary. **c** MDA-MB-231 and SNU-251-RR cells were treated with scrambled (Sc), BRCA1 exon 11 (E11) or intron 15 (I15)-targeting siRNA and BRCA1 protein expression assessed by Western blotting. **d** Cells were treated as in panel (**c**) and examined by IF for BRCA1 and RAD51 foci post 10 Gy IR. ***p < 0.001 compared to Sc-treated cells. See Supplementary Fig. 3e for representative images. **e** Cells were treated as in panel (**c**) and subject to increasing concentrations of rucaparib and colony formation assessed. Data are the mean ± SD of *n* = 3 biological replicates. Statistical significance was assessed by unpaired, two-tailed *t* tests.

Because the BRCT domain is required for protein interactions and several biological activities^15^, we examined the functionality of BRCA1-I15 in SNU-251 cells. Similar to BRCA1 full-length, BRCA1-I15 was located in the nuclear cellular fraction (Supplementary Fig. 3a)^37^. Furthermore, while BRCA1 BRCT mutant MDA-MB-436 cells lacked both BRCA1 and RAD51 γ-irradiation-induced foci (IRIF); SNU-251 parental cells demonstrated foci positivity, and the number of BRCA1 and RAD51 foci positive cells was elevated in SNU-251-RR cells (Fig. 4a and Supplementary Fig. 3b). SNU-251 parental and RR cells also exhibited HR activity, detected using the direct-repeat green fluorescent protein (DR-GFP) reporter system^19^ (Fig. 4b and Supplementary Fig. 3c−d). Additionally, BRCA1 intron 15-targeting siRNA depleted BRCA1 protein (Fig. 4c), reduced BRCA1 and RAD51 IRIF (Fig. 4d and Supplementary Fig. 3e), and sensitized SNU-251-RR cells to PARPi treatment, but had no effect on MDA-MB-231 cells (Fig. 4e and Supplementary Fig. 3f). These data indicate that the BRCA1-I15 protein isoform is functional and integral to RAD51 loading and PARPi resistance in SNU-251-RR cells.

### BRCTless isoforms avoid proteasomal degradation

**Fig. 5:**
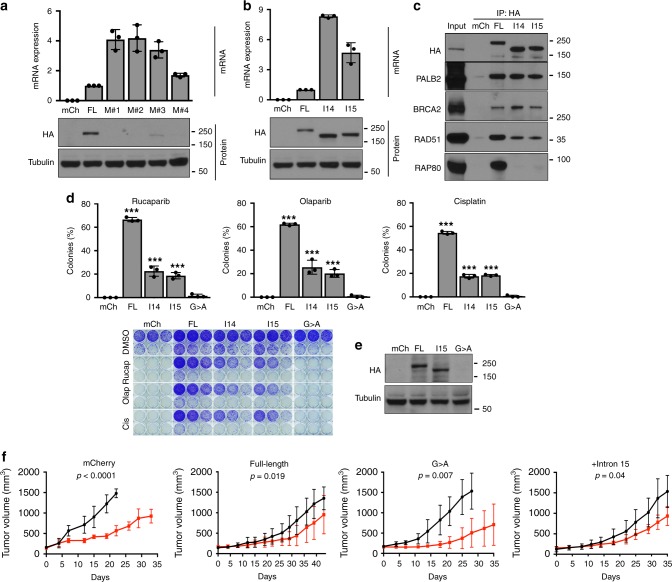
BRCA1-I15 is stable and hypomorphic. **a** MDA-MB-436 cells engineered to express mCherry (mCh), HA-BRCA1 full-length/wild-type (FL), or the HA-BRCA1 BRCT mutations (M) c.5251 C > T-(M#1), c.5263insC-(M#2), c.5277insTA-(M#3), c.5445 G > A-(M#4), that are endogenously present in HCC1395, HCC1937, MDA-MB-436 and SNU-251 *BRCA1* BRCT mutant cell lines, respectively; and were assessed for HA mRNA by qRT-PCR. HA expression normalized to FL expressing cells is shown (above). HA protein expression was assessed from the same cells by Western blotting (below). **b** MDA-MB-436 cells engineered to express mCh, BRCA1-FL, BRCA1-I14-(I14), and BRCA1-I15-(I15) were assessed as described in **a**. See Supplementary Fig. 4b. **c** Cells from panel **b** were subject to HA-immunoprecipitation with the indicated antibodies. **d** mCh, BRCA1-FL, BRCA1–14, BRCA1-I15, and BRCA1 c.5445 G > A (SNU-251 mutation) expressing MDA-MB-436 cells were seeded at decreasing densities in the presence of 20 nM rucaparib, 20 nM olaparib or 20 ng/ml cisplatin and assessed for colony formation. ***p < 0.001 compared to mCh cells. Below, representative plates. For panels **a**−**d**, data are the mean ± SD of *n* = 3 biological replicates. Statistical significance was assessed by unpaired, two-tailed *t* tests. **e** mCh, BRCA1-FL, BRCA1-I15, and BRCA1 c.5445 G > A expressing MDA-MB-436 tumor xenografts were harvested and assessed for HA expression by Western blotting. **f** Mice harboring mCh, BRCA1-FL, BRCA1-I15, and BRCA1 c.5445 G > A expressing MDA-MB-436 tumor xenografts were treated with vehicle (black line) or 200 mg/kg rucaparib bi-daily (red line) for 2 × 5 days with a 2-day interval. Mean ± SD. Tumor volumes are shown for five tumors implanted in five separate mice per cell line per treatment from the same experiment. A linear mixed effects model tested the difference in slopes of log- tumor volume, and *p* values shown are tests of differences in growth rates between vehicle and rucaparib-treated tumors. Moreover, differences-in-slopes (i.e. vehicle to PARPi differences) were compared using Tukey’s correction for multiple comparisons: BRCA1 5445 G > A vs. BRCA1-I15 (*p* *=* 0.02), BRCA1 5445 G > A vs. BRCA1-FL (*p* *<* 0.001), BRCA1-I15 vs. mCh (*p* *=* 0.04), and mCh vs. BRCA1-FL (*p* *=* 0.001), 5445 G > A vs. mCh (*p* *=* 0.99) or BRCA1-I15 vs. BRCA1-FL (*p* *=* 0.57). See Supplementary Data 1 file for more details.

To determine why a shorter 1570 aa BRCA1-I15 protein might be selectively expressed in place of a closer to full-length *BRCA1* c.5445 G > A generated 1814 aa protein, ectopic HA-tagged BRCT domain mutant BRCA1 proteins were expressed and subject to mRNA and protein expression analyses. While BRCT mutation-containing *HA-BRCA1* mRNA expression was readily detected, protein expression was low or undetectable (Fig. 5a). MG132 treatment increased the expression of BRCA1 BRCT mutant proteins (Supplementary Fig. 4a), indicating that mutations induced proteasomal degradation.

We hypothesized that due to the location of intron-coded stop codons, specifically intron 14- and intron 15-retaining mRNAs could generate BRCA1 isoforms that would retain the CC domain, but lack the entire BRCT domain (Supplementary Fig. 4b)^15^. Therefore, proteins should be capable of promoting PALB2-BRCA2-RAD51 loading^26,37^, as well as avoiding BRCT mutation-associated protein degradation^37^. To assess this possibility, we generated HA-BRCA1 constructs that contained aa’s and translation stop sites encoded by introns 14 and 15 (Supplementary Fig. 4b). Here, HA-BRCA1-I14 and -I15-expressing cells had similar mRNA and protein expression as full-length HA-BRCA1 (Fig. 5b). Thus, in contrast to BRCT domain mutant proteins, BRCA1-I14 and -15-containing proteins are stable and abundant. Furthermore, while BRCA1-I14 and -15 did not associate with BRCT domain interacting proteins, the CC domain enabled PALB2-BRCA2-RAD51 interaction (Fig. 5c). BRCA1-I14 and -15 were also capable of forming foci and supporting RAD51 IRIF, although to a lesser degree than full-length BRCA1 (Supplementary Fig. 4c).

In colony assays, BRCA1-I14- and -I15 expressing cells formed significantly more colonies in the presence of rucaparib, olaparib and cisplatin compared to mCherry and *BRCA1* c.5445 G > A mutant expressing cells (Fig. 5d). Similar observations were made in xenografts experiments, where differences between vehicle and PARPi treatments were more pronounced in mCherry and *BRCA1* c.5445 G > A, compared with BRCA1-full-length and BRCA1-I15 expressing tumors (Fig. 5e, f). Of note, PARPi’s are known to be moderately effective in the treatment of subsets of *BRCA1* wild-type cancers^56^, and we found that PARPi-treated BRCA1-full-length expressing tumors had a statistically significant growth delay compared to vehicle-treated tumors. Additionally, the vehicle-treated BRCA1-full-length expressing tumors’ growth rate was slower than other groups. Overall, BRCA1 BRCTless isoforms did not demonstrate the same level of activity as full-length BRCA1; nevertheless, they avoid proteasomal degradation and are hypomorphic, promoting RAD51 loading and PARPi resistance.

### *BRCA1* rearrangements occur in patient-derived tumors

**Fig. 6:**
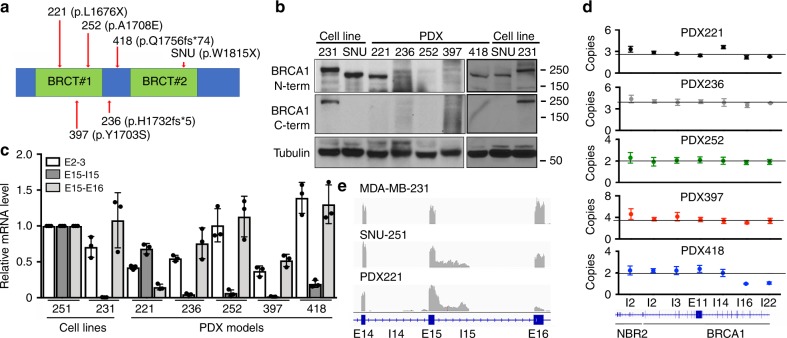
BRCA1-I15 is expressed in patient-derived tumors. **a** Cartoon showing the location of *BRCA1* mutations present in PDX models relative to *BRCA1* BRCT repeats. See Supplementary Table 2 for more information. **b** PDX tumors were examined for BRCA1 protein expression using N- or C-terminal-specific antibodies by Western blotting. MDA-MB-231 (231) and SNU-251 (SNU) cells are comparators. **c** *BRCA1* exons 2–3, intron 15, and exons 15–16 mRNA expression in the PDX samples measured by qRT-PCR. MDA-MB-231 (231) and SNU-251 (SNU) cells are included as comparators. **d** Copy number analyses as described in Fig. 2e. **e** *BRCA1* exons 15–16 reads detected using RNA-seq in PDX221 compared to MDA-MB-231, SNU-251-RR cells. Data are the mean ± SD of *n* = 3 biological replicates.

**Fig. 7:**
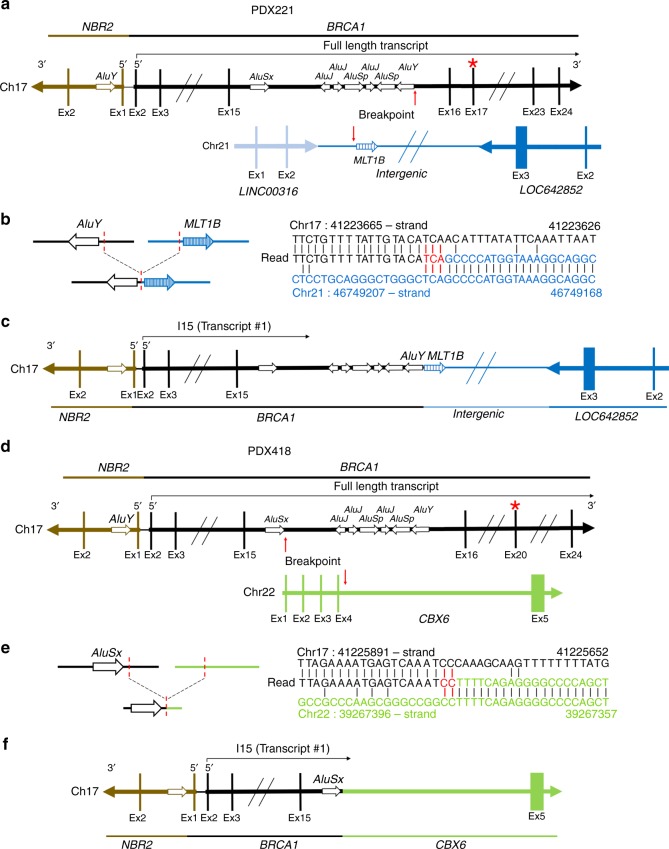
BRCA1 intron 15 translocations detected in PDX tumors. **a** Schematic depicting the non-rearranged *BRCA1* locus and chr:21q22.3 region. Chr:21q22.3 contains the long intervening/intergenic noncoding RNAs (lincRNAs) *LINC00316* and *LOC642852*. PDX221 has a c.5027delT mutation located in *BRCA1* exon 17 that is noted with a red asterisk. Chromosome breakpoints are indicated (red arrows). **b** The breakpoints and junction are depicted and are within 93 bp of an *AluY* sequence in *BRCA1* intron 15 and within 36 bp of a *MLT1B* sequence in chr21q22.3, dashed red line (see Supplementary Note 1). Right, reads detected by WGS that map to the indicated regions of *BRCA1* intron 15 and chr21 are shown. Sequence numbers are the genomic coordinates according to the UCSC genome browser (GRCh37/hg19 assembly). Red sequence represents a region of microhomology. The read sequence colors correspond to the relative chromosome regions shown (left). **c** Schematic showing the chr17:21 translocation detected PDX221 that results in transcription termination in intron 15 and generates the *BRCA1* intron 15 mRNA isoform. See Supplementary Fig. 5b−d and Supplementary Note 1 for more details. **d** Schematic depicting the non-rearranged *BRCA1* locus and chr:22q23.1 region. PDX418 has a c.5266dupC mutation located in *BRCA1* exon 20 that is noted with a red asterisk, and the breakpoints (red arrows) are indicated. **e** The breakpoints and junction are depicted and are within 117 bp of an *AluSx* sequence in *BRCA1* intron 15; however, a SINE element was not detected in the *CBX6* intron 4 sequence (see Supplementary Note 1). Right, reads detected by WGS that map to the indicated regions of *BRCA1* intron 15 and *CBX6* intron 4 are shown. Sequence numbers are the genomic coordinates according to the UCSC genome browser (GRCh37/hg19 assembly). Red sequence represents a region of microhomology. The read sequence colors correspond to the relative chromosome regions shown (left). **f** Schematic showing the chr17:22 translocation detected PDX418 that results in transcription termination in intron 15 and generates the *BRCA1* intron 15 mRNA isoform. See Supplementary Fig. 5b−d and Supplementary Note 1 for more details.

We set out to examine BRCA1-I15 expression in a panel of five unique patient-derived xenograft (PDX) models that harbored different *BRCA1* BRCT domain mutations (Fig. 6a and Supplementary Table 2)^57,58^. Here, PDX221 and PDX418 both generated BRCA1 proteins that had a similar gel migration pattern as SNU-251 cells, and were detectable with N-terminal but not C-terminal-specific BRCA1 antibodies (Fig. 6b). *BRCA1-I15* mRNA and protein expression was confirmed using qRT-PCR and mass spectrometry (Fig. 6c and Supplementary Fig. 5a). Interestingly, PDX418, but not PDX221, showed partial *BRCA1* gene duplication, ending in *BRCA1* intron 15 (Fig. 6d). RNA-seq analyses showed that PDX221 reads ended at an identical location as those detected in SNU-251-RR cells, suggesting the same IPA site (Fig. 6e). WGS analyses of PDX221 showed that a translocation t(17;21)(q21.31;q22.3) had occurred, where a sequence immediately adjacent to the *BRCA1* intron 15 *AluY* sequence was fused to an *ERV3* superfamily *MLT1B* element sequence located in an intergenic region of chromosome 21q22.3 (Fig. 7a and Supplementary Fig. 5b-d)^59^. Interestingly, the junction site had 3 bp of microhomology (Fig. 7b and Supplementary Note 1). This rearrangement is predicted to result in transcription termination within intron 15, producing BRCA1-I15 (Fig. 7c). Additionally, PDX418 showed a chromosome 17:22 translocation t(17;22)(q21.31;q23.1), with break sites immediately 3′ of the *AluSx* sequence in *BRCA1* intron 15, as well as a region that lacked designated SINE elements within intron 4 of the *CBX6* gene (Fig. 7d and Supplementary Fig. 5b-d). The junction site also demonstrated 2 bp of microhomology (Fig. 7e and Supplementary Note 1), potentially indicating a role of microhomology-mediated end joining (MMEJ) in generating the translocations detected in PDX221 and PDX418^60,61^. The rearrangement detected in PDX418 is also predicted to result in transcription termination within intron 15 and to produce BRCA1-I15 (Fig. 7f). Thus, *BRCA1* intron 15 rearrangements that generate translocations can be detected in chemo-refractory patient-derived tumors.

## Discussion

*BRCA1* BRCT domain mutations result in loss of tumor suppressor activity and are commonly found in patients with hereditary forms of breast and ovarian cancer. *BRCA1* BRCT domain missense and truncating mutations disrupt BRCT-BRCT repeat contacts and are structurally destabilizing^32–35^. BRCT mutant proteins that are incapable of folding correctly are subsequently susceptible to proteasomal degradation, and BRCA1 BRCT mutant protein expression is frequently low or undetectable in tumors and cell lines^21,39,40^. We previously found MDA-MB-436 cells, which harbor a *BRCA1* BRCT domain mutation, have undetectable BRCA1 protein expression, lack RAD51 foci, and demonstrated exquisite PARPi sensitivity^40^. In contrast, we report here that SNU-251 cells, which also harbor a disruptive BRCT domain mutation, have detectable BRCA1 protein expression, RAD51 foci, and are relatively insensitive to PARPi, requiring only a 2–3-week period for unperturbed cell growth in the presence of 1 μM rucaparib.

The BRCA1 BRCT domain is required for multiple protein interactions and activities^62–64^. In agreement, we found that BRCTless BRCA1 was less efficient compared with full-length BRCA1 at promoting RAD51 loading and PARPi resistance in an isogenic cell line system. However, BRCTless BRCA1 was partially proficient for RAD51 loading, and was capable of inducing PARPi resistance at a significantly greater level than the mCherry control. Therefore, we conclude BRCTless BRCA1 isoforms are hypomorphs, and promote PARPi resistance when abundantly expressed^62^. Importantly, loss of the entire BRCT domain is required to generate proteins that avoid proteasomal degradation^37^. Furthermore, BRCTless proteins are dependent on the CC domain for activity, and more severe truncations that are deficient for the CC domain lack any hypomorphic activity^38^.

Intron retention is a mechanism that regulates gene expression and proteomic diversity, having been extensively studied in organisms such as plants and budding yeast. In mammalian systems, the role of intron retention is more elusive, but several studies have shown that intron retention is a central component of gene expression programs during normal development, as well as in cancer^65,66^. Analyses of *BRCA1* mRNA indicated that SNU-251 cells likely express two dominant isoforms; the first being full-length, and the second intron 15-retaining. However, rather than epigenetic regulation, intron 15 was translated because of *BRCA1* gene rearrangements.

*BRCA1* gene *Alu*-mediated rearrangements are associated with pathogenic loss-of-function mutations^49,67–69^. In this study, we found *Alu*-mediated gain-of-function events that were coupled with restored DNA repair and chemotherapy resistance. *BRCA1* intron 15 has seven *Alu* sequences, accounting for ~50% of intronic DNA^50^, making this region particularly susceptible to rearrangements. The DNA repair mechanisms that contribute to generating *Alu*-mediated rearrangements may be inferred from mutational signatures at breakpoints^60,61^. We propose that NAHR contributed to the *Alu-Alu*-mediated rearrangement found in SNU-251 cells, while MMEJ may have participated in generating the translocations observed in PDX tumors^48,50,51,55^.

Overall, the BRCA1-I15 hypomorphic isoform was detected in SNU-251 cells as well as 2/5 of BRCT mutant PDX samples assessed, and all were derived from patients who had previously received DNA damaging chemotherapy^41,57,58^. Thus, *BRCA1 Alu*-mediated rearrangements represent an additional mechanism of PARPi/chemo-resistance, and could serve as a predictive biomarker of clinical response in patients with *BRCA1* BRCT mutant cancers.

## Methods

### Cell lines and reagents

SNU-251 cells were obtained from the Korean Cell line bank. All other cell lines were from ATCC. SNU-251 cells were cultured in the presence of 1 µM rucaparib until a resistant population emerged (SNU-251-RR). Cells were cultured in the absence of rucaparib for a minimum of 2 weeks before they were used for experiments. Cell lines tested negative for mycoplasma and identities were confirmed using short-tandem repeat (STR) profiling by IDEXX Bioresearch. Chemicals were purchased from Sigma-Aldrich unless stated otherwise. Clovis Oncology provided rucaparib. Olaparib and niraparib were purchased from Selleckchem. Cisplatin was from APP/Fresenius Kabai USA LLC.

### Colony formation assays

Cells were seeded at decreasing densities in six-well plates and maintained in vehicle or in the presence of 100 nM rucaparib, 100 nM olaparib, 100 nM niraparib or 50 ng/ml cisplatin until resistant colonies emerged. For siRNA treatments, exponentially growing cells were reverse transfected in 24-well plates, 24 h post transfection cells were treated with increasing concentrations of rucaparib for 72 h and re-plated in six-well plates for colony formation. Colony formation was assessed with crystal violet staining. Mean and standard deviation (SD) colony formation from three experiments was expressed as percentage of colonies relative to vehicle group. LC50 values (concentration required to reduce colony formation by 50% compared to vehicle) were calculated using GraphPad Prism software and used to compare fold changes in drug sensitivity.

### Immunoprecipitation, mass spectrometry and Western blotting

BRCA1 (EMD Millipore, catalog# OP92) antibody was used for immunoprecipitation of BRCA1 complexes from 2 mg of nuclear extract using Pierce Classic IP Kit (ThermoFisher Scientific, catalog# 26146) according to the manufacturer’s instructions. Nuclear extracts were derived using NE-PER Nuclear and Cytoplasmic Extraction Reagents (ThermoFisher Scientific, catalog# 78833) according to the manufacturer’s instructions. For peptide analyses, after immunoprecipitation, bands corresponding to BRCA1 were cut out and gel purified, digested using chemotrypsin and analyzed by LC-MS/MS. Proteins detected by Western blotting used the following antibodies: Tubulin (1:2000, Cell Signaling, catalog# 2148), N-terminal BRCA1, MS110 (1:500, EMD Millipore, catalog# OP92), C-terminal BRCA1, D9 (1:500, Santa Cruz Biotechnology, catalog sc-6954), HA, 6E2 (1:500, Cell Signaling, catalog# 2367), 53BP1 (1:1000, EMD Millipore, catalog# MAB3802), PARP1 (1:1000, Cell Signaling, catalog# 9542), BRCA2 (1:1000, Bethyl, catalog# A303-434A), PALB2 (1:1000, Bethyl, catalog# A301-247A), RAD51 (1:500, Santa Cruz Biotechnology, catalog sc-8349), RAP80 (1:1000, Bethyl, catalog# A300-763A). For assessment of protein stabilization, cells were incubated with either 10 µM MG132 or 10 µg/ml cycloheximide for 6 or 12 h or 500 nM HSP90 inhibitor (AT13387, Selleckchem) for 48 h. Cells were then collected and subjected to Western blotting. See Source Data file for uncropped Western blot images.

### Immunofluorescence and microscopy

Cells were pre-extracted in cytoskeleton buffer (10 mM PIPES pH 6.8, 100 mM NaCl, 300 mM sucrose, 3 mM MgCl_2_, 1 mM EGTA, 0.5% Triton X-100) for 5 min and followed by cytoskeleton stripping buffer (10 mM Tris-HCl pH 7.4, 10 mM NaCl, 3 mM MgCl_2_, 1% Tween 20, 0.5% Sodium deoxycholate) for 5 min on ice. Cells were then fixed with 4% formaldehyde and permeabilized by 1% Triton X-100 in phosphate-buffered saline (PBS). BRCA1 (1:2000, EMD Millipore, catalog# OP92), HA (1:1000, Covance, catalog# MMS-101R), RAD51 (1:1000, Genetex, catalog# GTX100469), γ-H2Ax (1:1000, R&D Systems, catalog# AF2288) and 53BP1 (1:10000, EMD Millipore, catalog# MAB3802) antibodies were followed by secondary antibodies conjugated to FITC or Texas Red (Jackson ImmunoResearch Laboratories). Cells were mounted in Vectashield containing DAPI (Vector Laboratories Inc.). We acquired immunofluorescence images using Nikon NIU Upright Fluorescent Microscope and generated images using Nikon NIS Elements software. For IR experiments, we routinely fixed cells 7 h after treatment with 10 Gy. For analyses, we counted a minimum of 200 cells per condition per cell line. Each experiment was carried out at least three times.

### Flow cytometric analysis

For cell cycle analysis, cells were harvested and fixed with 70% ethanol. Cells were washed with PBS and resuspended in 0.5 ml of FxCycle™ PI/RNase Staining Solution (ThermoFisher Scientific, catalog**#** F10797) and incubated at room temperature for 15 min. Data were acquired using BD FACScan Flow Cytometer and analyzed using FlowJo software. For cell death analysis, cells were collected 5 days after 1 µM rucaparib treatment and analyzed using PE Annexin V Apoptosis Detection Kit I (BD Biosciences, catalog**#** 55976) according to the manufacturer**’**s instructions. Data were acquired using BD LSR II Flow Cytometer and analyzed using FlowJo software.

### DR-GFP assay

In order to account for differences in transfection efficiency between cell lines, we replaced the puromycin cassette in the DR-GFP reporter with BFP^70^. The plasmid was pre-cut by I-SceI (NEB, catalog# R0694). Cells were seeded in 24-well plate and transfected with 0.5 µg pre-cut plasmid using 1.5 µl TransIT-2020 (Mirus, catalog# MIR 5400). Forty-eight hours later, cells were collected and analyzed by using BD LSR II Flow Cytometer. The HR efficiency was defined as the percentage of GFP-positive cells in BFP-positive population.

### Gene sequencing analyses and qRT-PCR

Genomic DNA was isolated from cells or tumors using the DNeasy Blood and Tissue kit (Qiagen, catalog**#** 69504). Total RNA was isolated from cell lines using RNeasy Plus Mini Kit (Qiagen, catalog**#** 74134). RT-PCR with a forward primer located in *BRCA1* exon 11 and reversed primers in exon 24 and intron 15 were used to detect WT and I15 *BRCA1* mRNA. BROCA sequencing was carried out to detect mutations in DNA repair genes^22,71^. Sanger sequencing was used to detect the *BRCA1* c.5445 G > A mutation in exon 23 and the potential mutations around exon 15/intron 15 splicing site. Primer sequences used in this study are listed in Supplementary Table 3.

### RNASeq

RNAseq was performed on HiSeq 2500 system using Truseq stranded mRNA library kit, Hiseq rapid PE cluster kit and HiSeq rapid SBS kit (Illumina,CA) according to the manufacturer’s manual. Briefly, 1 μg total RNAs from each sample were used to make Stranded mRNA-seq library. mRNAs were enriched twice via poly-T-based RNA purification beads, and subjected to fragmentation via a divalent cation method. The first strand cDNA was synthesized by SuperscriptII, followed by second strand synthesis. During second strand synthesis, the dUTP was used to replace dTTP, thereby the second strand was quenched during amplification. A single “A” nucleotide is added to the 3′ ends of the blunt fragments. Adapters with illumina P5, P7 sequences as well as indices were ligated to the cDNA fragment. Libraries were again purified using AmPure beads, and subject to a quality check using a bioanalyzer (Agilent) and quantified with Qubit (Invitrogen). Sample libraries were subsequently pooled and paired end reads at 105 bp generated for downstream bioinformatic analysis. Quality of the RNA-Seq reads were analyzed using FastQC: (http://www.bioinformatics.babraham.ac.uk/projects/fastqc). Reads were aligned to human genome (Hg38) using Tophat2 (Mapped reads to Hg38 - SNU-251-RR: 96 Million reads; MDA-MB-231: 75 Million reads; PDX-221: 78 Million reads). IGV was used to visually depict the genomic regions and sequencing reads.

### 3′ RACE

Rapid Amplification of cDNA Ends (RACE) was performed to identify the 3′ UTR of the I15 *BRCA1* isoform. cDNA was generated using SuperScript III First-Strand Synthesis System (ThermoFisher Scientific, catalog**#** 18080051) and oligo dT with the adapter sequence. The 3′ UTR sequence was determined by Sanger sequencing following two-round nested PCR. Primer sequences are listed in Supplementary Table 3.

### Whole-genome sequencing

WGS was performed by Novogene Corporation. A total amount of 1.0 μg DNA per sample was used as input material for the DNA sample preparations. Sequencing libraries were generated using NEBNext® DNA Library Prep Kit following the manufacturer’s recommendations and indices were added to each sample. The genomic DNA is randomly fragmented to a size of 350 bp by shearing, then DNA fragments were end polished, A-tailed, and ligated with the NEBNext adapter for Illumina sequencing, and further PCR enriched by P5 and indexed P7 oligos. The PCR products were purified (AMPure XP system) and resulted libraries were analyzed for size distribution by Agilent 2100 Bioanalyzer and quantified using real-time PCR. Libraries were sequenced on an Illumina platform according to an effective concentration and data volume. Burrows−Wheeler Aligner (BWA) was used to map the paired-end clean reads to the human reference genome (hg19). Primary and secondary mapping sites of discordant split-reads that map the breakpoint regions in BRCA1 loci were identified by parsing CIGAR flags of mapped reads. Integrative Genomics Viewer (IGV) was used to visually depict the genomic regions and sequencing reads. SINE elements were screened using CENSOR (https://www.girinst.org/censor/index.php).

### Copy number variation assay

Genomic DNA was isolated from cells or tumors using the DNeasy Blood and Tissue kit (Qiagen, catalog**#** 69504). TaqMan Copy Number Reference Assay, HumanRNase P (LifeTechnologies, catalog**#** 4403326) was used as an internal control and run with Taqman Universal master mix (ThermoFisher Scientific, catalog**#** 4304437). BRCA1 copy number was analyzed using Power SYBR Green master mix (Applied Biosystems, catalog**#** 4368702). The *BRCA1* copy number of each sample was determined by qPCR targeting different region spanning the *BRCA1* locus using RPE cells as a normalizer. The sequence of primers are listed in Supplementary Table 3.

### RNA interference and cDNA add back treatments

We purchased Hs_BRCA1_FlexiTube siRNA construct (constructs #9 - SI00299495, targeting exon 11), and AllStars Negative Control siRNA (scrambled control) from Qiagen. Custom-made *BRCA1* intron 15 targeting siRNA sequence: UCUGCUGUAUUGGAACAAAUU (Dharmacon), ∆11q targeting siRNA sequence: GUAUCAGGGUGAAGCAGCAUU (Dharmacon). Transfections were carried out according to standard protocols. mCherry and HA-BRCA1 cDNA were cloned into pENTR1A Gateway Entry vector and shuttled into a pDest-IRES-GFP Destination vector. To generate BRCA1 proteins which contained amino acids and translation stop sites encoded by introns 14 and 15(I14, I15), a small fragment corresponding to the specific intron retaining BRCA1 was first amplified and then used to replace the C-terminal region of *BRCA1* in a pENTR-HA-BRCA1-V5 (full-length) construct using StuI (I14), AleI (I15) and BsaAI sites. cDNAs were shuttled into pLenti-IRES-GFP Destination vectors using the LR Clonase system (ThermoFisher Scientific, catalog**#** 11791020). Lentiviral generation and infections were carried out according to standard protocols. Protein knockdown was routinely assessed 72 h post transfection. Cells infected with cDNAs were sorted for GFP positivity using the FACS Aria II cell sorter and routinely checked for GFP positivity to maintain stable cell lines.

### Xenograft treatments and analyses

MDA-MB-436 cells were subcutaneously implanted in 6-week-old female NSG mice. Treatment was initiated when tumors reached between 150 and 180 mm^3^. Rucaparib was administered at 150 mg/kg twice daily for 10 continuous days with a 2-day break after the first 5 days. Vehicle treatment consisted of 0.5% methylcellulose in water. Tumors were measured with calipers and tumor volumes calculated using the formula: (length × width^2^). Measurements were carried out every 3 days and mice euthanized when tumors reached 1500 mm^3^ in accordance with Institutional Animal Use and Care Committee (IACUC) guidelines of Fox Chase Cancer Center (FCCC). All work involving mice received ethical approval by the IACUC at FCCC.

### Patient-derived xenografts

Patient consent for tumor use in animals was completed under a protocol approved by the Vall d’Hebron Hospital Clinical Investigation Ethical Committee and Animal Use Committee. Mice were maintained and treated in accordance with institutional guidelines of Vall d’Hebron University Hospital Care and Use Committee. Tumors were subcutaneously implanted in 6-week-old female HsdCpb:NMRI-Foxn1nu mice (Harlan Laboratories). Animals were supplemented with 1 mmol/l estradiol (Sigma) in the drinking water. Upon xenograft growth, tumor tissue was reimplanted into recipient mice, which were randomized upon implant growth.

### Statistical analyses

Mouse tumor growth curve data were analyzed using linear mixed effects models. For each experiment, the differences in growth rates of log-transformed tumor volumes between vehicle and rucaparib treatments were calculated. We fit linear mixed-effects models with random intercepts and slopes to account for within-mouse correlation, and tested the interaction effects between treatment and time. We assessed whether there was a nonlinear effect of time by examining residual plots, and tested models which include a quadratic effect of time for improved fit using likelihood ratio tests. Reported *p* values are from tests of the interaction terms, interpreted as differences in growth rates between vehicle and rucaparib treatments. Moreover, for xenografts, we fit a single regression model containing all experimental conditions to test the three-way interaction of time × treatment × xenograft model. Similar to the other regression models for log-volume, we fit a linear mixed-effects model with random intercepts and slopes to account for within-xenograft correlation and included a quadratic effect of time. The additional three-way interaction allowed the rates of growth (slopes) to vary by treatment and mouse model. After fitting the model, we tested all pairwise comparisons (contrasts) of the differences-in-slopes between treatment conditions, adjusting for multiple comparisons via Tukey’s method (see Supplementary Data 1 for more details). For all other experiments, mean and SD values are shown (GraphPad Software). *p* < 0.05 was considered statistically significant and statistical tests are indicated in the figure legends. Asterisks indicate statistically significant *p* values. There were similar variances between statistical groups compared.

### Reporting summary

Further information on research design is available in the Nature Research Reporting Summary linked to this article.

## Supplementary information

Supplementary Information

Description of Additional Supplementary Files

Supplementary Data 1

Supplementary Data 2

Reporting Summary

## Data Availability

All data supporting the findings in this study are available within the paper, Supplementary Information and Source Data files. RNA-Seq and WGS data are available at the Sequence Read Archive (SRA) database under Bioproject ID PRJNA588424. The mRNA sequence of the *BRCA1* intron 15-containing isoform described in this study has been deposited in the NCBI GenBank nucleotide database with GenBank accession number MN577637.

## Source data

Source Data
