# Supplementary material for: BRCA1 intronic Alu elements drive gene rearrangements and PARP inhibitor resistance
Source: Nat Commun. 2019 Dec 11;10:5661. doi: 10.1038/s41467-019-13530-6 (PMC6906494; doi:10.1038/s41467-019-13530-6)
Supplement: Supplementary file 1 — Supplementary Information [file 41467_2019_13530_MOESM1_ESM.pdf]

SUPPLEMENTARY INFORMATION

BRCA1 intronic *Alu* elements drive gene rearrangements and PARP inhibitor resistance

Wang et al.

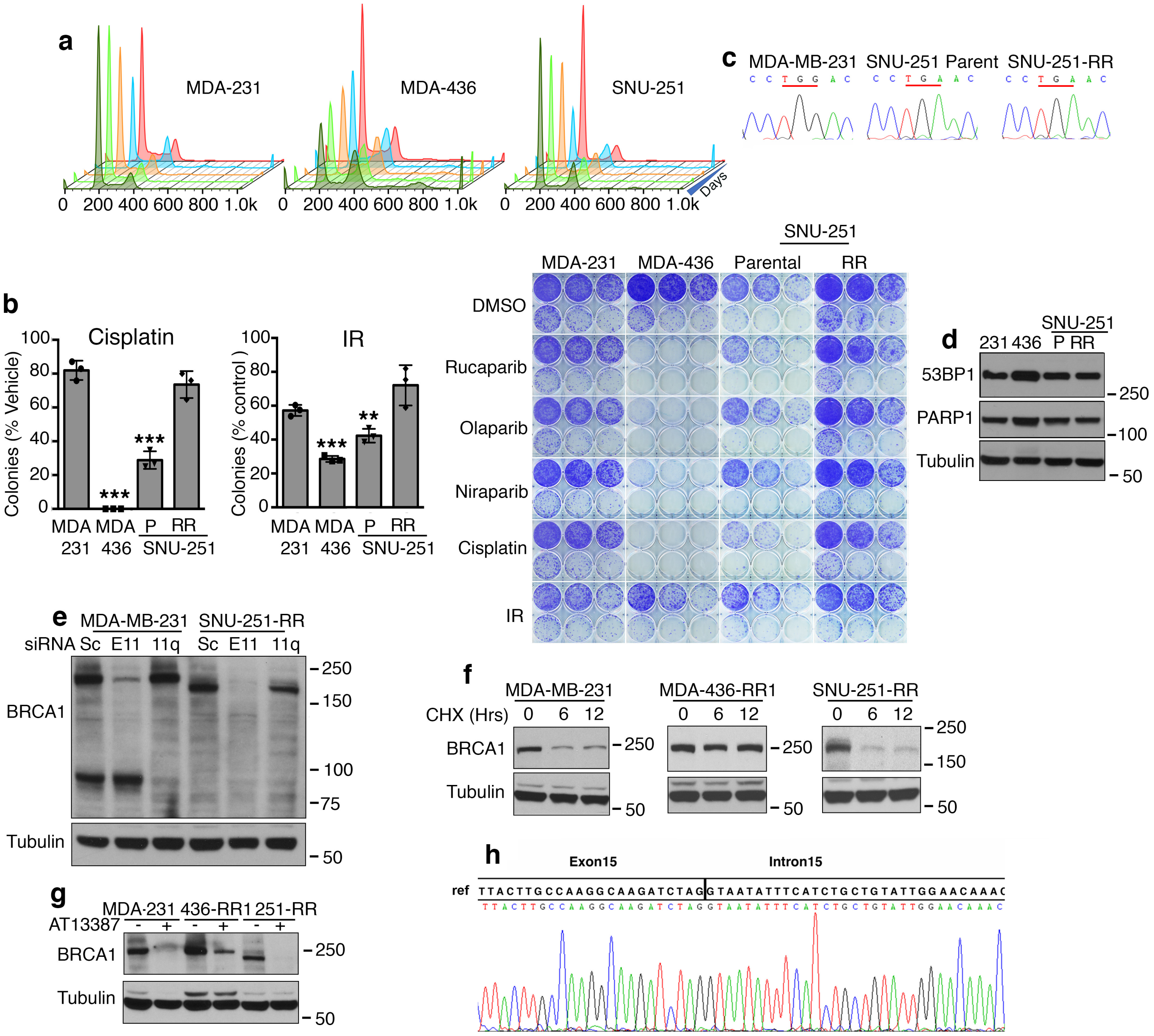

Supplementary Figure 1. Additional characterization of SNU-251 cells.

(a) MDA-MB-231, MDA-MB-436 and SNU-251 cells were maintained in the presence of 1 µM rucaparib and collected for analyses every 24 hours. Cells were stained with propidium iodide and cell cycle profiles were determined by flow cytometry. Representative cell cycle profiles are shown, *n*=3 biological replicates. (b) MDA-MB-231, MDA-MB-436, SNU-251 parental (P), SNU-251 rucaparib resistant (RR) cells were seeded at decreasing densities in the presence of either vehicle or 50 ng/ml cisplatin or 2 Gy IR and colonies counted 2-

weeks post-seeding. Cell survival was expressed as a percentage of vehicle-treated cells. Data are the mean ± S.D. of $n = 3$ biological replicates. Statistical significance was assessed by unpaired, two-tailed t-tests. *** $p < 0.001$, compared to MDA-MB-231. Representative plates are shown for data presented in Fig. 1c. (c) Sanger sequencing was carried out and electropherograms are presented showing retention of the *BRCA1* c.5445G>A mutation in SNU-251 and SNU-251-RR cells. BROCA sequencing was also performed, which covers the entire *BRCA1* gene, and confirmed the absence of secondary reversion mutations in SNU-251-RR cells, see Supplementary Data 2 file for BROCA sequencing results. (d) 53BP1 and PARP1 expression were assessed by Western blotting. (e) MDA-MB-231 and SNU-251-RR cells were treated with scrambled (Sc), BRCA1 exon 11 (E11) or BRCA1-Δ11q (11q) targeting siRNA and lysates collected for Western blotting. (f) MDA-MB-231, MDA-MB-436-RR1 that were previously characterized to be dependent on HSP90 for folding[1], and SNU-251-RR cells were treated with cycloheximide (CHX) for 0, 6, or 12 hours and lysates collected for Western blotting. In line with our previous work, inhibition of translation with CHX treatment did not impact HSP90-associated stabilized BRCA1 protein levels in MDA-MB-436 RR-1 cells[1]. (g) Cell lines described in f were treated with HSP90 specific inhibitor AT13387 (500 nM) for 48 hours and lysates collected for Western blotting. Interestingly, all BRCA1 isoforms, including full-length/wild-type, showed HSP90 dependency. (h) Sanger sequencing was performed on DNA from SNU-251-RR cells. Electropherogram compared to the hg38 reference sequence shows the *BRCA1* exon 15 intron 15 junction. No splice site mutations were present in SNU-251 or SNU-251-RR cells.

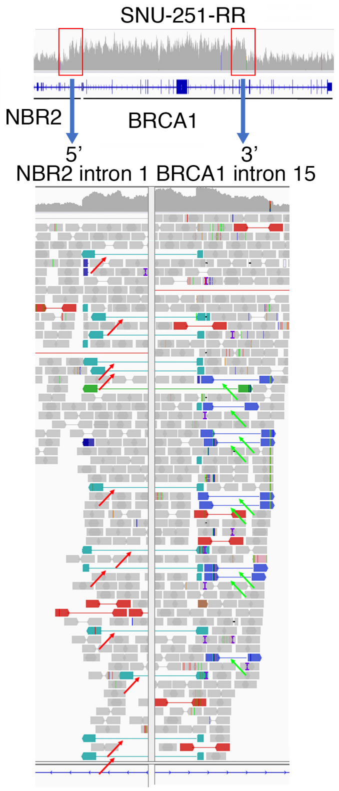

Supplementary Figure 2. WGS analyses of *BRCA1* in SNU-251-RR cells.

Above, Integrative Genomics Viewer (IGV) snapshot of WGS reads spanning *NBR2* and *BRCA1* genes on chromosome 17 detected in SNU-251-RR cells. Red box indicates the amplified 5' and 3' termini. *Below*, the 5' and 3' ends of the amplified *BRCA1* region is magnified to show individual reads. The amplified region starts (5') in *NBR2* intron 1 (Chr17: 41281775) and ends (3') in *BRCA1* intron 15 (Chr17: 4122418). Gray reads map 100% with hg19. Teal and blue flagged reads indicate single reads that map to both the sequence where the read is aligned, as well as additional sequences at alternative genomic locations. Red arrows and green arrows highlight reads with overlapping sequences that were used for deconvolution. See Fig. 3 for more details.

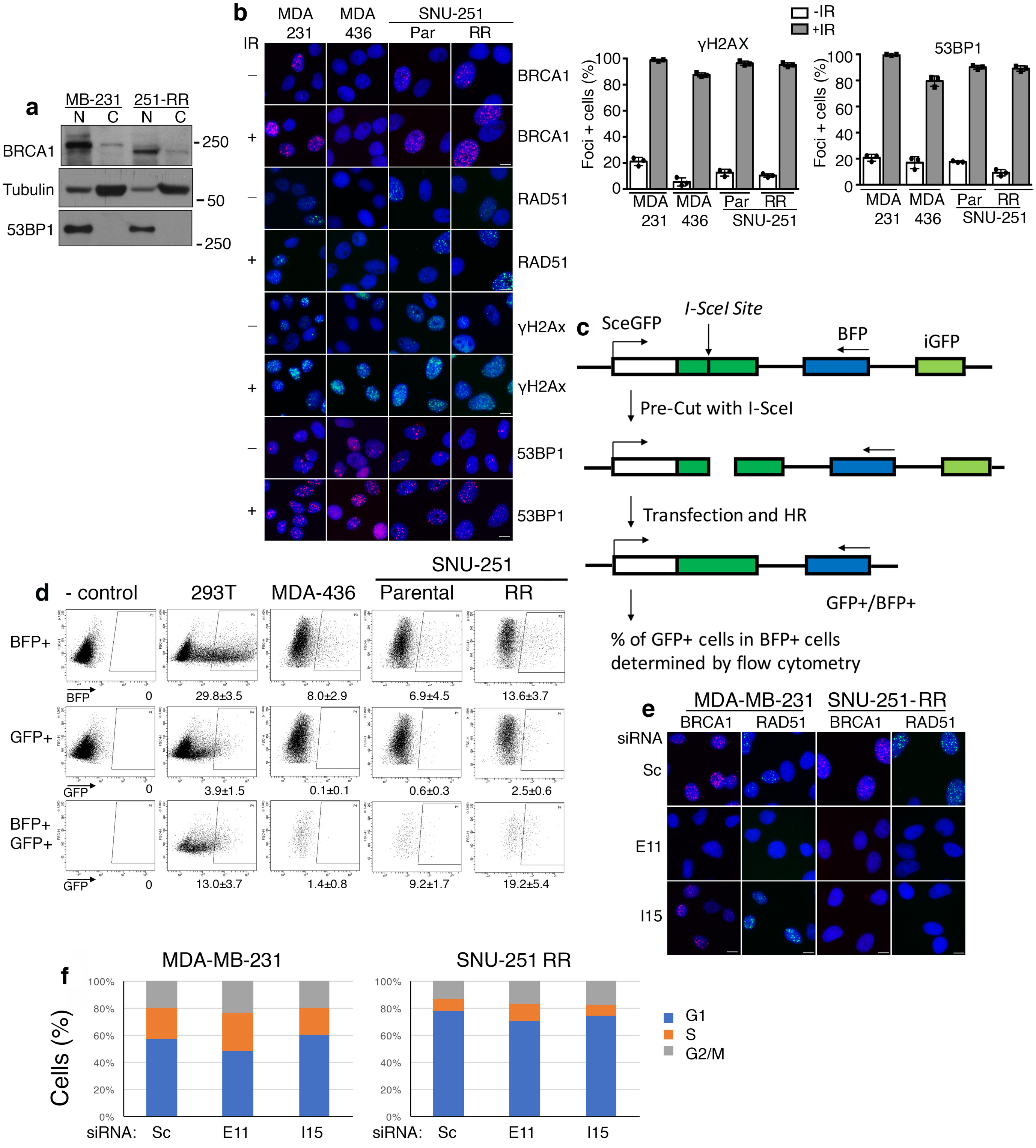

Supplementary Figure 3. Assessment of HR in SNU-251 cells.

(a) Nuclear (n) and cytoplasmic (c) extracts were generated and BRCA1 protein expression measured. 53BP1 and tubulin were measured as controls for nuclear and cytoplasmic separations, respectively. Of note, BRCT domain mutations have previously been shown to result in the protein being located in the cytoplasmic

compartment. There are no known localization signals in the BRCT domain; rather, BRCT mutations reduce nuclear import due to altered protein folding and degradation by cytoplasmic proteasome complexes. We and others have previously shown more extensive C-terminal deletions, occurring prior to the BRCT repeats, restores nuclear BRCA1 expression, as proteins folded correctly[2,3]. Of note exon 11 contains nuclear localization signals are retained in BRCA1-BRCTless proteins[3,4]. (b) Representative images from Fig. 4a as well as 53BP1 and γH2AX controls showing DNA damage foci was present across all cell lines. Scale bar is 10 μm. (c) Cartoon of modified DR-GFP assay. In order to exclude the difference caused by transfection efficiency between the cell lines, we replaced the puromycin cassette with BFP[5]. The plasmid was pre-cut by I-SceI and transfected into cells followed by flow cytometry. The HR efficiency was defined as the percentage of GFP positive cells in BFP positive population. (d) Representative flow cytometry graphs showing BFP and GFP status as well the mean ± S.D. of $n = 3$ biological replicates gated. See Fig. 4b. (e) Representative images from Fig. 4d. Scale bar is 10 μm. (f) Cell cycle distributions of cells from siRNA treatments described in Fig. 4c-e. Changes in RAD51 foci resulting from BRCA1 siRNA treatment were not a result of diminished S/G2/M phase cells. Data throughout are mean ± S.D. $n = 3$ biologically replicates.

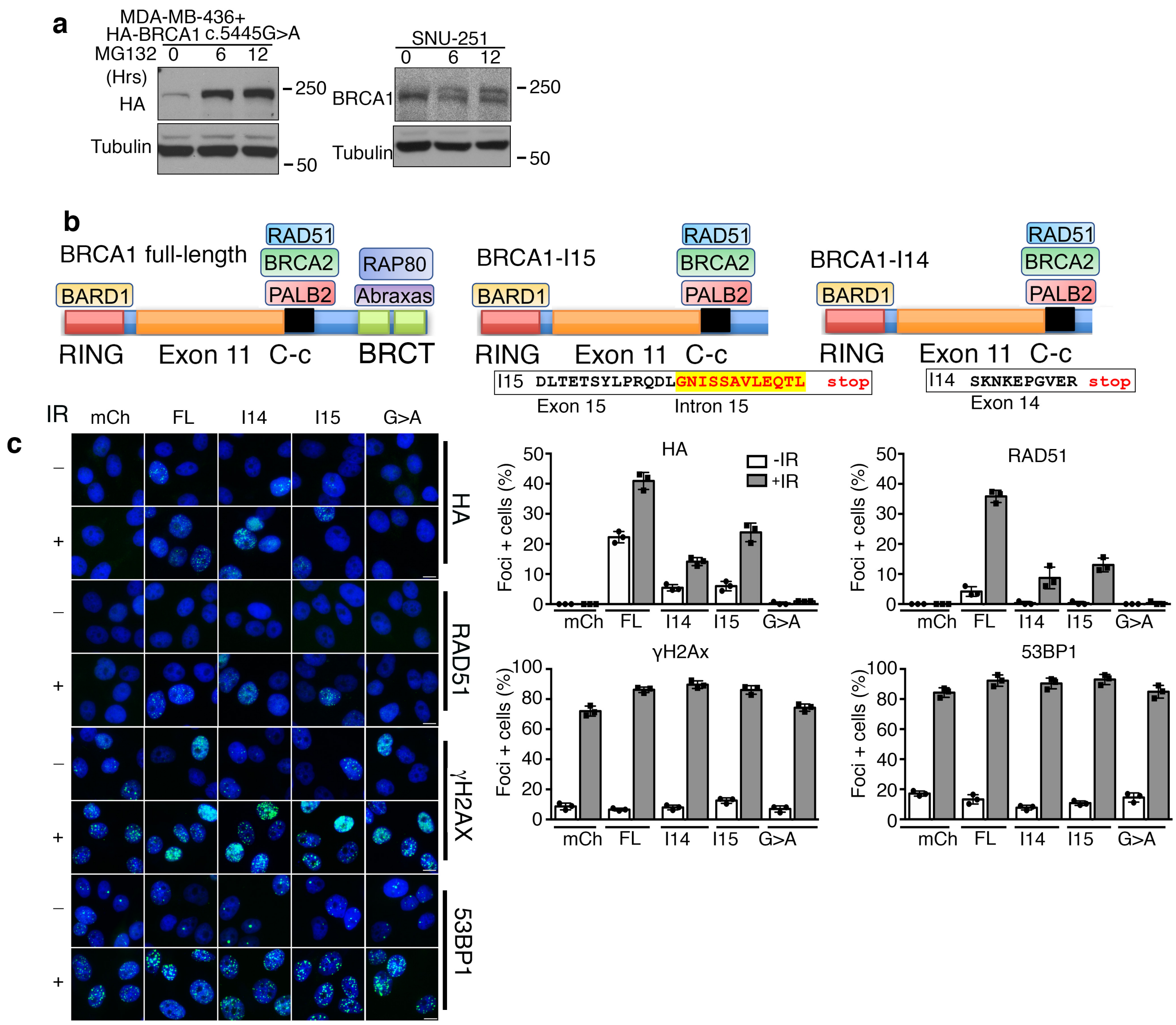

Supplementary Figure 4. Characterization of BRCA1 intron-containing proteins.

(a) MDA-MB-436 cells engineered to express HA-BRCA1 c.5445G>A and SNU-251 cells were treated with MG132 for 0, 6, and 12 hours and ectopic HA or endogenous BRCA1 assessed by Western blotting. MG132 resulted in marked increase in ectopic c.5445G>A HA-BRCA1 protein levels in MDA-MB-436 cells. Similarly, when SNU-251 cells were treated with MG132, we observed a BRCA1 protein band migrating above the intron 15 containing isoform, potentially the *BRCA1* c.5445G>A associated protein product that is 1814 aa in length. However, we were unable to immunoprecipitate enough protein to confirm this by mass spectrometry. Thus, *BRCA1* BRCT domain mutations, including the mutation present in SNU-251 cells, induce proteasomal

degradation, likely resulting from protein folding defects. (b) Cartoon of full-length BRCA1 showing functional domains relative to BRCA1-intron 14 and BRCA1-intron 15 generated stop codons and protein isoforms. BRCA1 amino acid sequences generated from intron 14 and intron 15 through to intron-induced stop codons are depicted. Intron 14 codes for an immediate stop codon after exon 14. Intron 15 codes for 12 amino acids followed by a stop codon. (c) Representative images and HA, RAD51, γ-H2AX and 53BP1 foci from MDA-MB-436 cells expressing the indicated constructs in the presence or absence of IR. mCherry (mCh), Full-length (FL), intron 14 (I15), intron 15 (I15), c.5445G>A (G>A). Scale bar is 10 μm. Data are the mean ± S.D. $n = 3$ biological replicates.

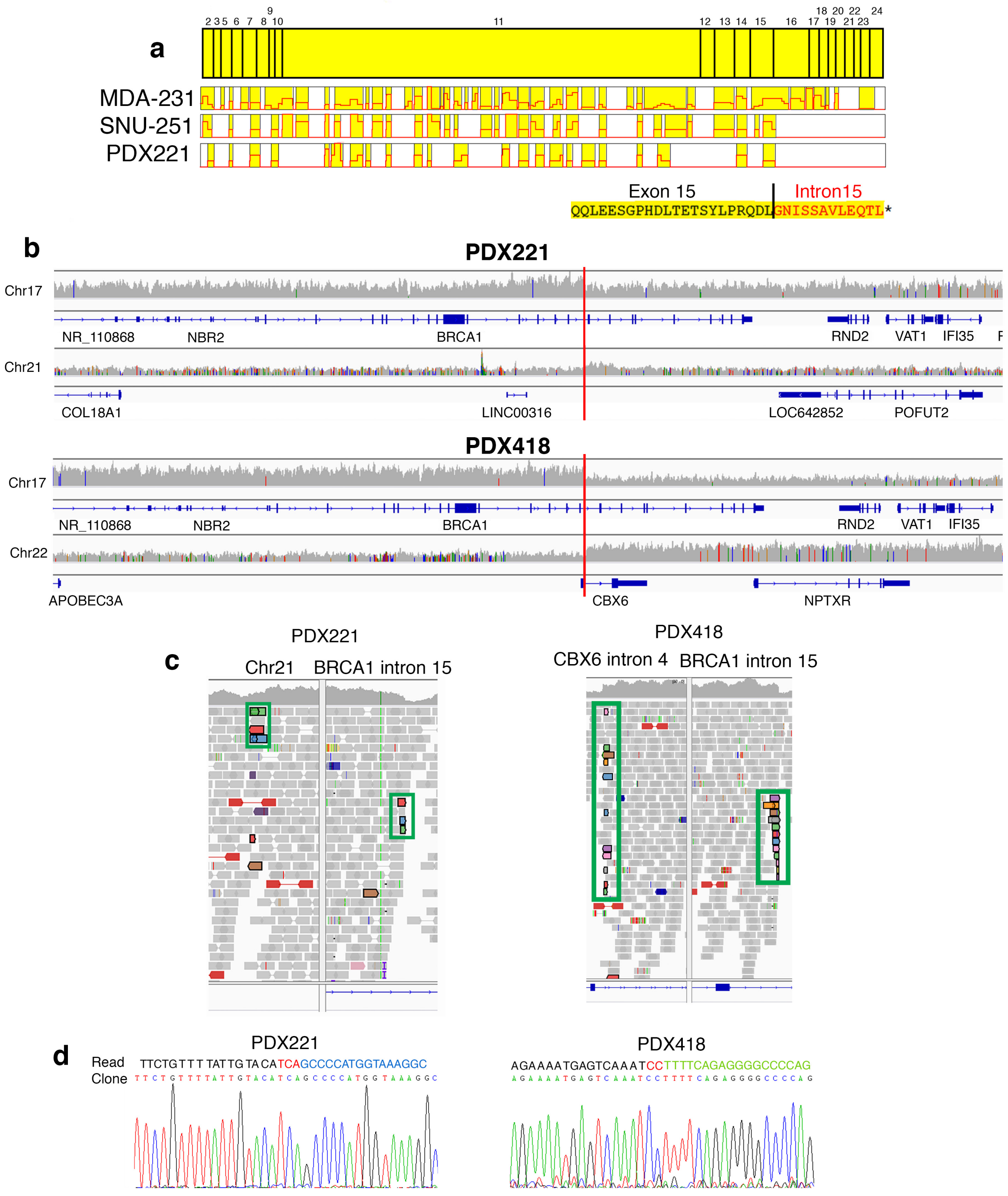

Supplementary Figure 5. Proteomic and WGS analyses of *BRCA1* in PDX tumors.

(a) BRCA1 was immunoprecipitated from PDX221 lysates and subject to mass spectrometric analyses as described in Fig. 1. The identical BRCA1-I15 peptide identified in SNU-251-RR cells was also found in PDX221. (b) IGV of reads that mapped to both chromosome 17 and 21 regions where breakpoints are located in PDX221; and chromosome 17 and 22 where breakpoints are located in PDX418. Red line indicates breakpoints,

gray bars on y axis correspond to the number of reads detected on the particular chromosome location. (c) View of individual reads detected in PDX221 are shown that mapped to both chromosome 17 and 21; as well as individual reads detected in PDX418 that mapped to both chromosome 17 and 22. Green box highlights the single split reads. (d) The t(17;21)(q21.31;q22.3) and t(17;22)(q21.31;q23.1) translocation junctions in PDX221 and PDX418, respectively, were cloned and sequences confirmed by Sanger sequencing.

Supplementary Table 1. Cell line information.

Cell line	Disease	BRCA1 (HGVS)	BRCA2 (HGVS)
**MDA-MB-231**	TNBC	WT	WT
**SNU-251**	OvCa	c.5445G>A	WT
**MDA-MB-436**	TNBC	c.5277+1G>A	WT

TNBC – triple negative breast cancer; OvCa – ovarian cancer. Of note, the BRCA1 wild-type allele was undetectable in SNU-251 and MDA-MB-436 cells. See BROCA appendix table in Supplementary Data 2 file for more information.

Supplementary Table 2. PDX information.

PDX	Disease	BRCA1 (HGVS)	Protein predicted	Therapies prior to establishing PDX	PDX Olaparib
**221**	TNBC	c.5027delT	p.Leu1676*	1. NeoAdj doxorubicin + cyclophosphamide + 5FU → paclitaxel	PD
**236**	TNBC	c.5194-12G>A	p.His1732Phefs*5	1. NeoAdj doxorubicin + cyclophosphamide + docetaxel; 2. paclitaxel + gemcitabine; 3. capecitabine; 4. pegylated irinotecan	PD
**252**	TNBC	c.5123C>A	p.Ala1708Glu	1. NeoAdj epirubicin + docetaxel; 2. carboplatin + gemcitabine; 3. lurbinectedin (PM01183)	PD
**397**	OvCa	c.5108A>C	p.Tyr1703Ser	1. carboplatin + paclitaxel; 2. carboplatin + pegylated liposomal doxorubicin;	PD
**418**	ER+BC	c.5266dupC	p.Gln1756Profs*74	1. paclitaxel + gemcitabine; 2. talazoparib; 3. carboplatin + gemcitabine → carboplatin; 4. capecitabine; 5. eribulin; 6. pegylated liposomal doxorubicin; 7. docetaxel	PD

PD – progressive disease/growth is similar to vehicle treated tumors

ER+BC – estrogen receptor+ breast cancer

Supplementary Table 3. Primer sequences

RT-PCR primers for detecting Wild-type and I15 BRCA1 isoforms	
BRCA1 exon11 F	GATGATGGTGAAATAAAGGAAGATACTA
BRCA1 exon24 R	GATCTGGGGTATCAGGTAGGTGTC
BRCA1 intron15 R	ATCTTGGCTCACCGCAACC
qRT-PCR primers for detecting Wild-type and I15 BRCA1 isoforms	
BRCA1 exon2/3 F	TTATCTGCTCTTCGCGTTGAAG
BRCA1 exon2/3 R	TTGTGGAGACAGGTTCCTTGAT
BRCA1 exon15/intron15 F	ATTAAGGTTGTTGATGTGGAGGAG
BRCA1 exon15/intron15 R	AGTGTTTGTTCCAATACAGCAGATG
BRCA1 exon15/16 F	GAATAGAAACTACCCATCTCAAGAGGA
BRCA1 exon15/16 R	CAGGTAAGGGGTTCCCTCTAGAT
BRCA1 exon18/19 F	AAATATTTTCTAGGAATTGCGGGA
BRCA1 exon18/19 R	TCTTTCTTTAATAGACTGGGTCACCC
RPLP0 F	CCCATTCTATCATCAACGGGTACAA
RPLP0 R	CAGCAAGTGGGAAGGTGTAATCC
HA F	CCTACGACGTGCCCGACTA
BRCA1 exon2 (used for HA) R	ATGGGACACTCTAAGATTTTCTGCA
Primers used to detect the mutations in exon 23 and exon 15/intron 15	
BRCA1 intron22 F	AGGTGTGAGCATCGCTTGAAC
BRCA1 intron23 R	CCCACAGCCTCTGGCAACT
BRCA1 intron14 F	ACAGGGCTGTATAGCAGTCCAGTA
BRCA1 intron15 R	GGCATGAGCAGTGGCTGTAAT
Primers used in 3'RACE	
oligo dT with adapter	GAGGACTCGAGCTCAAGCCGCGTCGACTAGTAC$(T)_{18}$
1st Round F (*BRCA1* exon 13)	ACTCAGCAGAGGGATACCAT
1st Round R	GAGGACTCGAGCTCAAGC
2nd Round F (*BRCA1* exon 15)	CACAGTTGCTCTGGGAGTCTTC
2nd Round R	AGCCGCGTCGACTAGTAC
Primers used in copy number variation assay	
NBR2-I2F	CCAGAGCCACAGAACATGTAAG
NBR2-I2R	GGGATAGAGGTGAGATCCAAAC
BRCA1-I2F	GCTCTATGAGAGCAGGCTTTATT
BRCA1-I2R	ACCAAGGCATTCCTGAGATTAG
BRCA1-I3F	TTGTTATTTGGTGTGAGATAGGAGT
BRCA1-I3R	GCCCAGGCCAAAGATGATTA
BRCA1-Ex11F	TAGCAAGGAGCCAACATAACAGAT
BRCA1-Ex11R	CTTATTCCATTCTTTTCTCTCACACAG
BRCA1-I14F	CAGGTCTCACCCATCTTCAATC
BRCA1-I14R	TTTAAAGGGCCTCAGAGGTTAAT
BRCA1-I16F	GCATCACCTTGGGTCCTTAAT
BRCA1-I16R	GGGTGGTAAACTTCTCAGGATG
BRCA1-I22F	AATATTCTCTCCTGTGAGCAAGAC
BRCA1-I22R	AGTTCTCAAATCCTTACCCATCC

Primers used to make I14, I15 *BRCA1* cDNA	
BRCA1 intron13 F	TTTCATGTCTGACACTCATGTCTTG
BRCA1 intron14 R	GGAGGCTAAGGTGGGAGGAT
BRCA1 exon11 F	GATGATGGTGAAATAAAGGAAGATACTA
BRCA1 intron15 R	ATCTTGGCTCACCGCAACC

SUPPLEMENTARY NOTE 1. gDNA sequences

gDNA sequence at *BRCA1* locus breakpoints in SNU251-RR Cells

Uppercase: BRCA1 Exon15 (chr17)
Lowercase: BRCA1 intron15 (chr17)
Alu element
Uppercase: reversed insertion of BRCA1 Exon15 (chr17)
Lowercase: reversed insertion of intron15 (chr17)
Alu element
Lowercase: reversed insertion of NBR2 intron1 (chr17)
Alu element
random insertion
167 bp sequence duplicated at both breakpoints

GTCATCCCCTTCTAAATGCCCATCATTAGATGATAGGTGGTACATGCACAGTTGCTCTGGGAGTCTTCAGAATAGAAACTA
CCCATCTCAAGAGGAGCTCATTAAGGTTGTTGATGTGGAGGAGCAACAGCTGGAAGAGTCTGGGCCACACGATTTGACG
GAAACATCTTACTTGCCAAGGCAAGATCTAGgtaatatttcatctgctgtattggaacaaacactttgattttactctgaatcctacataaagatatt
ctggttaaccaacttttagatgtactagtctatcatggacacttttgttatacttaattaagcccactttagaaaaatagctcaagtgttaatcaaggtttacttgaa
aattattgaaactgttaatccatctatattttaattaatggtttaactaatgattttgaggatgagggagtcttggtgtactctaaatgtattatttcaggccaggc
atagtggctcacgcctgtaatcccagtactccaggaggccgaggcaggtggatcagctgaggtcaggagttcaagacctgtctggccaacatggtgaaaccct
gtctctactaaaaatacaaaaaaattaactgggtgtgctagtgcatgcccgtaatcctagctactctggaggctgaggcagcagaatcacttgaacccgggag
gcggaggttgcggtgagccaagatcacaccactgcactccagtctgggtgacagagcaagactccatctcaaaaaatatatatatatatatacacacatat
attttatttcaactgttagacaagagtccaaaggccaaagaataaagttttaggccagtcctttattagaaaatgagtcaaatcccaaagcaagtttttttatga
gttaatgaatataaatgactacatattttatgccttaaaaatcacttttaatgaatggtgttttatggcttgtaaatcagagttttaatcagtaaagaaagtttttaa
tcctcaaaaacacgttatcataaaagacactgtttggcatcaaatgtggtatttggccatgttcattagggtcattttaggaatctcatacattctacttagctatg
cttaattcctgataccatggcattttctgaaatgtttcaaggatgacatctctgctgtttttaatttggtaatgatatctgctgatttattaagtgaaaaaagtaatg
gtgtcattaccttggatgaagaaacaaaaataaagcatttgccacatttttcaacttttttttcctttcttacaaaattgctataagctcattgcccccaaattggac
aatatagggaataaaaaagataatttggggtggggttagacacgggtcttgttatgttgccgaggctggtctctaactcctggcctcatgcaatcttcctaccttg
gcctcccaaagtgctgggattataggtgtgagccacttcaccaagctgagatgccacctcttaaaagataaaataaggacagattacagccactgctcatgcc
tgtaatctcagtactttgggaggccaaggtgggagaattgctcgaggccaagagttcaagaccagcctgggcaatgtagcgagacctgatctctataaaaagt
aaaaaaaaaaaaaaaactagctggggccaggcgtggtgggtggcttacgcctgtaatcccagcactttgggaggccgaggcgggcagatcacctgaggtcaa
gaggtcgagaccagtctgaccaacatggagaaaccctgtctctactaaaaatacaaaaaattagccgggcgtggtggtgcatgcctgtaatcccagctactgg
ggaggctgaagcaggagaatcgctcgaacccaggaggcggaggttgcgctgagccgagattgcgccattgcactccagcctgggcaacaagagtgaaactc
cgtctcaaaaaagagtacgggtaatattttttttttgagaagggtctcggtctgtcatccaggctgcagtgcagtggcacaaacatggctcacctgcagccttg
acctcctggactcaagtgatcttcctgtctcagcctcctgagtagctgggaccacaggtgtttgccaccttccagctgattttttttttttttgagatggagtttcatt
cttgttgcccaggctagagtgcaatggcgcgatctcagctcactgaaacctctgcctcccaggttcaagcgattctcctgcctcagcctcccgagtagctgggac
tacaggcataagccaccaagcctggctaattttgtatttttagtagagacagggtttctccatattggtcaggttggtcctgaactcctgccctcaggtgatctgcc
cgcctcggcctcccaaagtgctgggattacaggcgtaagccacccaccacgcctggccccagctagtttttttttttttacttttatagagatcaggtctcgct
acattgcccaggctggtcttgaactcttggcctcgagcaattctcccaccttggcctcccaaagtactgagattacaggcatgagcagtggctgtaatctgtcctt
attttatcttttaagaggtggcatctcagcttggtgaagtggctcacacctataatcccagcactttgggaggccaaggtaggaagattgcatgaggccaggag
ttagagaccagcctcggcaacataacaagacccgtgtctaaccccaccccaaattatctttttattccctatattgtccaatttgggggcaatgagcttatagca
attttgtaagaaaggaaaaaaaagttgaaaaatgtggcaaatgctttatttttgtttcttcatccaaggtaatgacaccattactttttcacttaataaatcagca
gatatcattaccaaattaaaaacagcagagatgtcatccttgaaacatttcagaaaatgccatggtatcaggaattaagcatagctaagtagaatgtatgagat
tcctaaaatgaccctaatgaacatggccaaataccacatttgatgccaaacagtgtcttttatgataacgtgtttttgaggattaaaactttctttactgattaaa
actctgatttacaagccataaaacaccattcattaaaagtgatttttaaggcataaaatatgtagtcatttatattcattaactcataaaaaaaacttgctttggg

atttgactcattttctaataaaggactggcctaaaactttattctttggcctttggactcttgtctaacagttgaaataaaatatatgtgtgtatatatatatatatat
atttttgagatggagtcttgctctgtcacccagactggagtgcagtggtgtgatcttggctcaccgcaacctccgcctcccgggttcaagtgattctgcctca
gcctccagagtagctaggattacgggcatgcactagcacacccagttaatttttttgtatttttagtagagacagggtttcaccatgttggccagacaggtcttga
actcctgacctcagctgatccacctgcctcggcctcctggagtactgggattacaggcgtgagccactatgcctggcctgaaataatacatttagagtacacca
agactccctcatcctcaaaatcattagttaaaccattaattaaaatatagatggattaacagtttcaataattttcaagtaaaccttgattaacacttgagctattt
ttctaaagtgggcttaattaagtataacaaaagtgtccatgatagactagtacatctaaaagttggttaaccagaatatctttatgtaggattcagagtaaaatc
aaagtgtttgttccaatacagcagatgaaatattacCTAGATCTTGCCTTGGCAAGTAAGATGTTTCCGTCAAATCGTGTGGCCCAGAC
TCTTCCAGCTGTTGCTCCTCCACATCAACAACCTTAATGAGCTCCTCTTGAGATGGGTAGTTTCTATTCTGAAGACTCCCAG
AGCAACTGTGCATGTACCACCTATCATCTAATGATGGGCATTTAGAAGGGGATGAC-------------------------aggaacaaactc
tggacacatcatctttaagaactgtaccactcaccgtgagggtccgcaacttcattcttgaagtcagtgagaccaagaacccaccaatttcggacacaagaaca
tccggcttctactgatggaaggccacgactcctcagtagaaagagggcccaggcaaagaaaatgagagagagtccttggggtggtgacactatcttgggaac
atggagtgctaggaaacgataactggaagactaggatgaaatctgcaagttaaaaaatgacactacccatatatccttctcatcccattctcctttttcgagac
cccgttaaagagatcttggttagggacctttggttttggaaaggcactgtttctcctttttgtcaaacactcaactttcaggccacactttttttttttttcgagac
aaagtctcgctttctcaaagaccagcccaggctggtcttgaactcctgagctcaagcgatcctcctgccttggcctcccaaagtgctgggattacaggcatgagc
caccactcccggcctcagcttggataacttggcttgtacaggcctctggtggagacagtaaccaaagttcgggttgccaggtttgtttcattaaagggattttgtt
gaggctgaaagaaaaaaaaatcgatgtggaggaaaagctaacttgtgccaagacaaggtgagtcagtcacatggacttaacaataatgtcaattatgaaagc
tgaaggcctaggacccagctatctgactctactggctgtagacctaaggagcaggtagggttaaccatagctttcgtgtttacacacgttcaagattaatgctctt
gctgaaagaatttttttacatgccagccaaagagagttccttatggagacctggatatggtccagctaagctagaatctcccacttcctgttttctcttagttggcc
caccagctggaggagctccagaatatctaaattaatttccaattgtgtaatttgagaaaaagagacgaagattaatttccctaacgagtagggaagtgtgtttct
tttttttcttttttttttgagactgagtctcgctctgtcgcccagcctgaagtgcagtggcacgatcgcggctagtgcactgcgaactctgtctcccgggttcacgcc
attctcctgcctcagcctcccgagtagctgggactacaggcacccgccatcacgcccagctaatttttttgtgtttttagtagagatggggtttcaccgcgttagc
caggatgacctcgatctcctgacctcgtgatctgccttcctcggcctcccaaagtgctgggattataggcgtgagtcactgtgcctggcttcaaaatggccaagg
tgggagaattgctcgaggccaagagttcaagaccagcctgggcaatgtagcgagacctgatctctataaaaagtaaaaaaaaaaaaaaactagctggggcc
aggcgtggtgggtggcttacgcctgtaatcccagcactttgggaggccgaggcgggcagatcacctgagggcaggagttcaggaccaacctgaccaatatgg
agaaaccctgtctctactaaaaatacaaaattagccaggcttggtggcttatgcctgtagtcccagctactcgggaggctgaggcaggagaatcgcttgaacct
gggaggcagaggtttcagtgagctgagatcgcgccattgcactctagcctgggcaacaagaatgaaactccatctcaaaaaaaaaaaaatcagctggaag
gtggcaaacacctgtggtcccagctactcaggaggctgagacaggaagatcacttgagtccaggaggtcaaggctgcaggtgagccatgtttgtgccactgca
ctgcagcctggatgacagaccgagacccttctcaaaaaaaaaaatattacccgtactctttttttgagacggagtttcactcttgttgcccaggctggagtgcaat
ggcgcaatctcggctcagcgcaacctccgcctcctgggttcgagcgattctcctgcttcagcctccccagtagctgggattacaggcatgcaccaccacgcccg
gctaatttttgtatttttagtagagacagggtttctccatgttggtcagactggtctcgacctcttgacctcaggtgatccgcccaccttggcctcccaaagtgctg
ggattacaggcctgagccaccgcacccggcctgtactcttattctttaataataaaatatttctgtgtttctttagtcattttacataaactttatttatttatttattt
ttatttatttattttttgagacggagtctcgctctgttgcccaggctggaatgcaatggctcaatctcagctcactgcaagctctgcctcccgggtacacgccattc
ccctgcctcagcctccctagtagccgggactacaggcgcccgccaccacgcccagctaattttttttttgtattttcagtagagacagggtttcactgtgttagcc
aggatggtcttgatctcctgacctcgtgatccacccgtctcggcctcccaaagtgctgggattacaggtgtgagccaccgtgctcggcccataaacttttattttt
aaaataatgtcatgataaataatattgcttaggtgtctttaatatattagtaacatttctgttttattgtacatcaacatttatattcaaattaatgggtgaagagta
ctccattggactaggtatatcgtaatttaatctcctattattggacaactacattgtttctaaaattatactattcctatgactaaacctttgcatatatcttttatctc
cctaggatatatttctaaaactagcattgttgactgaaagtgtaaatacgtgttaaggtgtttgctacataatgccatatttcctttttaggaaactaagctactttg
gatttccaccaacactgtattcatgtacccatttttctcttaacctaactttattggtctttttaattcttaacagagaccagaactttgtaattcaacattcatcgttg
tgtaaattaaacttctcccattcctttcag

gDNA sequence at *BRCA1* locus breakpoint in PDX221

Uppercase: BRCA1 Exon15 (chr17)
Lowercase: BRCA1 intron15 (chr17)
Alu element
Lowercase: non-coding region (chr21)
MLT1B element
tca -microhomology at junction between BRCA1 and chr21

GTCATCCCCTTCTAAATGCCCATCATTAGATGATAGGTGGTACATGCACAGTTGCTCTGGGAGTCTTCAGAATAGAAACTA
CCCATCTCAAGAGGAGCTCATTAAGGTTGTTGATGTGGAGGAGCAACAGCTGGAAGAGTCTGGGCCACACGATTTGACG
GAAACATCTTACTTGCCAAGGCAAGATCTAGgtaatatttcatctgctgtattggaacaaacactttgattttactctgaatcctacataaagatatt
ctggttaaccaacttttagatgtactagtctatcatggacacttttgttatacttaattaagcccactttagaaaaatagctcaagtgttaatcaaggtttacttgaa
aattattgaaactgttaatccatctatattttaattaatggtttaactaatgattttgaggatgagggagtcttggtgtactctaaatgtattatttcaggccaggc
atagtggctcacgcctgtaatcccagtactccaggaggccgaggcaggtggatcagctgaggtcaggagttcaagacctgtctggccaacatggtgaaaccct
gtctctactaaaaatacaaaaaaattaactgggtgtgctagtgcatgcccgtaatcctagctactctggaggctgaggcagcagaatcacttgaacccgggag
gcggaggttgcggtgagccaagatcacaccactgcactccagtctgggtgacagagcaagactccatctcaaaaaatatatatatatatatacacacatat
attttatttcaactgttagacaagagtccaaaggccaaagaataaagttttaggccagtcctttattagaaaatgagtcaaatcccaaagcaagtttttttatga
gttaatgaatataaatgactacatattttatgccttaaaaatcacttttaatgaatggtgttttatggcttgtaaatcagagttttaatcagtaaagaaagtttttaa
tcctcaaaaacacgttatcataaaagacactgtttggcatcaaatgtggtatttggccatgttcattagggtcattttaggaatctcatacattctacttagctatg
cttaattcctgataccatggcattttctgaaatgtttcaaggatgacatctctgctgtttttaatttggtaatgatatctgctgatttattaagtgaaaaaagtaatg
gtgtcattaccttggatgaagaaacaaaaataaagcatttgccacattttcaacttttttttcctttcttacaaaattgctataagctcattgcccccaaattggac
aatatagggaataaaaaagataatttggggtggggttagacacgggtcttgttatgttgccgaggctggtctctaactcctggcctcatgcaatcttcctaccttg
gcctcccaaagtgctgggattataggtgtgagccacttcaccaagctgagatgccacctcttaaaagataaaataaggacagattacagccactgctcatgcc
tgtaatctcagtactttgggaggccaaggtgggagaattgctcgaggccaagagttcaagaccagcctgggcaatgtagcgagacctgatctctataaaaagt
aaaaaaaaaaaaaaactagctggggccaggcgtggtgggtggcttacgcctgtaatcccagcactttgggaggccgaggcgggcagatcacctgagggca
ggagttcaggaccaacctgaccaatatggagaaaccctgtctctactaaaaatacaaaattagccaggcttggtggcttatgcctgtagtcccagctactcggg
aggctgaggcaggagaatcgcttgaacctgggaggcagaggtttcagtgagctgagatcgcgccattgcactctagcctgggcaacaagaatgaaactccat
ctcaaaaaaaaaaaaaatcagctggaaggtggcaaacacctgtggtcccagctactcaggaggctgagacaggaagatcacttgagtccaggaggtcaag
gctgcaggtgagccatgtttgtgccactgcactgcagcctggatgacagaccgagacccttctcaaaaaaaaaaatattacccgtactctttttttgagacggag
tttcactcttgttgcccaggctggagtgcaatggcgcaatctcggctcagcgcaacctccgcctcctgggttcgagcgattctcctgcttcagcctccccagtagc
tgggattacaggcatgcaccaccacgcccggctaatttttttgtatttttagtagagacagggtttctccatgttggtcagactggtctcgacctcttgacctcaggt
gatccgcccaccttggcctcccaaagtgctgggattacaggcctgagccaccgcacccggcctgtactcttattctttaataataaaatatttctgtgtttctttag
tcattttacataaactttatttatttatttatttttatttatttattttttgagacggagtctcgctctgttgcccaggctggaatgcaatggctcaatctcagctcac
tgcaagctctgcctcccgggtacacgccattcccctgcctcagcctccctagtagccgggactacaggcgcccgccaccacgcccagctaatttttttttttgtatt
ttcagtagagacagggtttcactgtgttagccaggatggtcttgatctcctgacctcgtgatccacccgtctcggcctcccaaagtgctgggattacaggtgtgag
ccaccgtgctcggcccataaactttatttttaaaataatgtcatgataaataatattgcttaggtgtctttaatatattagtaacatttctgttttattgtacatcag
ccccatggtaaaggcaggcagaggatgggggctgttaggggctgagcggtgtcccccaaatccatgcatcaaagccctaacagccaggaccccagaaggtg
actgtatttggagacagcttttaaagaggtgattaaggtaaaattcagtccttagggcaggccctgatccagtctgcctggggtccttataaggggagggagga
cacagacacacggaggggtggccctgcgaggacccagggaggagacgccgcccacaagccaaggagaggctgcgggaggaaccggcctgccacacggg
gacctgggacgtccagcctccaggacgcagagaaacaacggtctgctgaggaagccgcccggcatgcagcatttgtgctggcagccctgagaaaacttgaat
aggatgctccgctgctggcatgagagctgttttcctgcaaggctgggcaactcctccaccgctggcgggtggaaaatggggcggccgccacgtgcctggtttatc
ccagagaggtgaggacctgggtacgcgcaagcacacaagcctgtgtggcagtgtccactgtggcaatgccaggacaatgatggctggaaaggacccgaagt
gccgccctggtaatggggtgacagacgggaacatctgaggcaggaggcggtgctcagatactggaccacattgaggcctagctaacacagggcaggggcac

aggtagctttccatgaggcacgccctccagtgcgccatgtcagtttacggttgccacggcaatacctgggcattactgccccttttccatggcaactacccgacca
cccaagagttactactccttccctagaaggagtgtccacctcacacaaggaccgtactggggcggcgtccagaaggagtgtccacctcacacaaggaccgtgc
tggggcggcgtccagaaggagtgtccacctcacacaaggaccgtgctggggcggcgtccgagaagtgtggggctgtctgcggccttgagggcctcgtgggcac
agtgcagacttggacacaggtggcaggactgggcgctcggagctgaggacgggtccaggagatgccgacaccagctaagctcctgctgtgagaccccgaccc
cggggcgttcaggaactccacccttttctgatcgcatcaccctcatggacagcctgcaagtccagtgacgttgtctccatttcacagatggagaggctgaggcttg
gtgccgagggcttcccacacatctgggagtgggagctgttctcaggccaagccaccctgggtccctgggaactcactccatcctgtgtgattccctgtgtgtcccc
tg

<u>gDNA sequence at *BRCA1* locus breakpoint in PDX418</u>

Uppercase: BRCA1 Exon15 (chr17)
Lowercase: BRCA1 intron15 (chr17)
Alu element
Lowercase: CBX6 intron4 (chr22)
cc -microhomology between BRCA1 and CBX6

GTCATCCCCTTCTAAATGCCCATCATTAGATGATAGGTGGTACATGCACAGTTGCTCTGGGAGTCTTCAGAATAGAAACTA
CCCATCTCAAGAGGAGCTCATTAAGGTTGTTGATGTGGAGGAGCAACAGCTGGAAGAGTCTGGGCCACACGATTTGACG
GAAACATCTTACTTGCCAAGGCAAGATCTAGgtaatatttcatctgctgtattggaacaaacactttgattttactctgaatcctacataaagatatt
ctggttaaccaacttttagatgtactagtctatcatggacacttttgttatacttaattaagcccactttagaaaaatagctcaagtgttaatcaaggtttacttgaa
aattattgaaactgttaatccatctatattttaattaatggtttaactaatgattttgaggatgagggagtcttggtgtactctaaatgtattatttcaggccaggc
atagtggctcacgcctgtaatcccagtactccaggaggccgaggcaggtggatcagctgaggtcaggagttcaagacctgtctggccaacatggtgaaaccct
gtctctactaaaaatacaaaaaaattaactgggtgtgctagtgcatgcccgtaatcctagctactctggaggctgaggcagcagaatcacttgaacccgggag
gcggaggttgcggtgagccaagatcacaccactgcactccagtctgggtgacagagcaagactccatctcaaaaaatatatatatatatatacacacatat
attttatttcaactgttagacaagagtccaaaggccaaagaataaagttttaggccagtcctttattagaaaatgagtcaaatcctttcagaggggccccagct
gggcagggggtggttgtgagcccccccagaagcccctgatggcaggcctcagccagcatcagcttcaacaagagggttttgggactggggataatcctccctg
ttgaaaccaggggctggggatgcctttagggctccaggaaagggggtggctgaggcaggtgggggatgcccatgggaagggggcatttgtttgagctgttatg
actgtgctgggagttggaacccagggggagagtgagtggggtcccacctcctggcagggcacaaggagaggatttgggggaccacctgcctccctgcccccca
gcttcccccgttcagtctttcaggatttgttcttcatggctcccctcctttcttggggatccaacttgaatttgacctcaaatcattttactttgaatttatcacccagg
ctgggccactaactttagggctctgagcctcagtttccccccactcccggccatttctgggggatggggatgtgggagtgagagcatagcatattagtctctgcta
gggagggaagaacttccagaggaagccatcttggaggatttgggtttttatttattttgacaaggaaaaacagagtctgggaaaggaagggcaagatggccc
taggtggcaggcacggctgcaggtgggactcagtgggcccccctgtcttcctgtagtgctacggatgcctcagggcccccctcccacccgacagccggtgactttgt
gggaggactctttttcggagaagagaagcagaagtgaaagaaatgttggtttccagaatttcatatttttataaccccccaccctcaagcgacgcacatcctagg
ggatccagggagtaaccttaaccctgatgcttctcttggggagggatgggcggagcagctggaaggctggtgggctgtgtgacagcctctcagtgcagctggg
gcaccctctagcggcgaagcgagtatgaccatgagcattgatacacagaacgcctttttttcttgatagttcttttggttcctgagctggctgtgggcctaataca
gggctgagcccccacatggattatctcctttaattctttcaacagttccaagaagtaaggagtattatccccatttcacagataaaaaatcaaggcccaggaagg
gaaagtgacttacccgaggtccagcttcctggcttcatcctgtctccttttcttcatcttgtgccttgagtagtccatgtcaggttaaagggggaaggcagacacac
acaggaaactatgaaggctgcagagttggcgtggcaggggatagagacacagatagtaggactttttttatctggaccctaagaaacaggtagcctgagagtg

Supplementary References

1 Johnson, N. *et al.* Stabilization of mutant BRCA1 protein confers PARP inhibitor and platinum resistance. *Proc Natl Acad Sci U S A* **110**, 17041-17046 (2013).

2 Rodriguez, J. A., Au, W. W. & Henderson, B. R. Cytoplasmic mislocalization of BRCA1 caused by cancer-associated mutations in the BRCT domain. *Exp Cell Res* **293**, 14-21 (2004).

3 Nacson, J. *et al.* BRCA1 Mutation-Specific Responses to 53BP1 Loss-Induced Homologous Recombination and PARP Inhibitor Resistance. *Cell reports* **24**, 3513-3527 e3517 (2018).

4 Wang, Y. *et al.* The BRCA1-Delta11q Alternative Splice Isoform Bypasses Germline Mutations and Promotes Therapeutic Resistance to PARP Inhibition and Cisplatin. *Cancer Res* **76**, 2778-2790 (2016).

5 Pierce, A. J., Johnson, R. D., Thompson, L. H. & Jasin, M. XRCC3 promotes homology-directed repair of DNA damage in mammalian cells. *Genes Dev* **13**, 2633-2638 (1999).
