# Supplementary material for: BRCA1 intronic Alu elements drive gene rearrangements and PARP inhibitor resistance
Source: Nat Commun. 2019 Dec 11;10:5661. doi: 10.1038/s41467-019-13530-6 (PMC6906494; doi:10.1038/s41467-019-13530-6)
Supplement: Supplementary file 2 — Description of Additional Supplementary Files [file 41467_2019_13530_MOESM2_ESM.docx]

**Description of Additional Supplementary Files**

**File Name: Supplementary Data 1**

**Description:** Statistical analyses of mouse tumor growth curve data in Fig. 5f.

**File Name: Supplementary Data 2**

**Description:** Summary of BROCA sequencing in MDA-MB-231, MDA-MB-436, SNU-251 parental and SNU-251-RR cells.
