# Supplementary material for: BRCA1 intronic Alu elements drive gene rearrangements and PARP inhibitor resistance
Source: Nat Commun. 2019 Dec 11;10:5661. doi: 10.1038/s41467-019-13530-6 (PMC6906494; doi:10.1038/s41467-019-13530-6)
Supplement: Supplementary file 5 — Reporting Summary [file 41467_2019_13530_MOESM5_ESM.pdf]

NCOMMS-18-08095B
Corresponding author(s): Neil Johnson
Last updated by author(s): Oct 17, 2019

Reporting Summary

Nature Research wishes to improve the reproducibility of the work that we publish. This form provides structure for consistency and transparency in reporting. For further information on Nature Research policies, see Authors & Referees and the Editorial Policy Checklist.

Statistics

For all statistical analyses, confirm that the following items are present in the figure legend, table legend, main text, or Methods section.

n/a	Confirmed	
☐	☒	The exact sample size (n) for each experimental group/condition, given as a discrete number and unit of measurement
☐	☒	A statement on whether measurements were taken from distinct samples or whether the same sample was measured repeatedly
☐	☒	The statistical test(s) used AND whether they are one- or two-sided<br>*Only common tests should be described solely by name; describe more complex techniques in the Methods section.*
☒	☐	A description of all covariates tested
☐	☒	A description of any assumptions or corrections, such as tests of normality and adjustment for multiple comparisons
☐	☒	A full description of the statistical parameters including central tendency (e.g. means) or other basic estimates (e.g. regression coefficient) AND variation (e.g. standard deviation) or associated estimates of uncertainty (e.g. confidence intervals)
☐	☒	For null hypothesis testing, the test statistic (e.g. F, t, r) with confidence intervals, effect sizes, degrees of freedom and P value noted<br>*Give P values as exact values whenever suitable.*
☒	☐	For Bayesian analysis, information on the choice of priors and Markov chain Monte Carlo settings
☒	☐	For hierarchical and complex designs, identification of the appropriate level for tests and full reporting of outcomes
☒	☐	Estimates of effect sizes (e.g. Cohen's d, Pearson's r), indicating how they were calculated

Our web collection on statistics for biologists contains articles on many of the points above.

Software and code

Policy information about availability of computer code

Data collection	Cellquest Pro v5.2, BD FACSDiva v7
Data analysis	BD FACSDiva v7, Flowjo v10, Integrative Genomics Viewer v2.5.3, GraphPad Prism v6, RNA-Seq reads FastQC: (http://www.bioinformatics.babraham.ac.uk/projects/fastqc). SINE elements - CENSOR (https://www.girinst.org/censor/index.php).

For manuscripts utilizing custom algorithms or software that are central to the research but not yet described in published literature, software must be made available to editors/reviewers. We strongly encourage code deposition in a community repository (e.g. GitHub). See the Nature Research guidelines for submitting code & software for further information.

Data

Policy information about availability of data

All manuscripts must include a data availability statement. This statement should provide the following information, where applicable:

- Accession codes, unique identifiers, or web links for publicly available datasets
- A list of figures that have associated raw data
- A description of any restrictions on data availability

RNA-seq data are in the process of being deposited in the GEO. mRNA sequence have been submitted to Genbank. The authors declare that all other data supporting the findings of this study are available within the paper and its supplementary information and source data files upon reasonable request.

Field-specific reporting

Please select the one below that is the best fit for your research. If you are not sure, read the appropriate sections before making your selection.

☒ Life sciences ☐ Behavioural & social sciences ☐ Ecological, evolutionary & environmental sciences

For a reference copy of the document with all sections, see nature.com/documents/nr-reporting-summary-flat.pdf

Life sciences study design

All studies must disclose on these points even when the disclosure is negative.

Sample size	Sample sizes were chosen based on our own results from previous experiments or reported data sets.
Data exclusions	No data were excluded from the analyses.
Replication	All in vitro experiments were biologically/independently replicated a minimum of 3 times (n = 3). The in vivo xenograft studies were performed once with 5 mice per treatment group. The Western blots from the in vivo xenograft tumors were performed once. The RNA-seq and WGS experiments were performed once.
Randomization	For the in vivo xenograft studies, mice were randomized before treatment with 5 mice per treatment group.
Blinding	No blinding was performed.

Reporting for specific materials, systems and methods

We require information from authors about some types of materials, experimental systems and methods used in many studies. Here, indicate whether each material, system or method listed is relevant to your study. If you are not sure if a list item applies to your research, read the appropriate section before selecting a response.

Materials & experimental systems

n/a	Involved in the study	
☐	☒	Antibodies
☐	☒	Eukaryotic cell lines
☒	☐	Palaeontology
☐	☒	Animals and other organisms
☒	☐	Human research participants
☒	☐	Clinical data

Methods

n/a	Involved in the study	
☒	☐	ChIP-seq
☐	☒	Flow cytometry
☒	☐	MRI-based neuroimaging

Antibodies

Antibodies used	Antibodies, with corresponding catalog numbers and manufacturers, are described in Methods under headings Immunoprecipitation, mass spectrometry and western blotting and Immunofluorescence and microscopy.
Validation	Antibodies were validated by the manufacturers. Appropriate positive and negative controls were used to ensure specificity.

Eukaryotic cell lines

Policy information about cell lines

Cell line source(s)	SNU-251 (Korean Cell line bank),<br>MD-MBA-436 (ATCC),<br>MD-MBA-231 (ATCC)
Authentication	All cell lines were authenticated by STR profiling by IDEXX Bioresearch.
Mycoplasma contamination	All cell lines were tested for mycoplasma contamination.
Commonly misidentified lines<br>(See ICLAC register)	No commonly misidentified cell lines were used.

Animals and other organisms

Policy information about studies involving animals; ARRIVE guidelines recommended for reporting animal research

Laboratory animals	6-week-old female NSG mice and 6-week-old female HsdCpb:NMRI-Foxn1nu mice were used.
Wild animals	No wild animals were used.
Field-collected samples	No field-collected samples were used.
Ethics oversight	All studies with animals were approved by Fox Chase Cancer Center and the Vall d'Hebron Hospital Clinical Investigation Ethical Committee and the Institutional Animal Use and Care Committee.

Note that full information on the approval of the study protocol must also be provided in the manuscript.

Flow Cytometry

Plots

Confirm that:

☒ The axis labels state the marker and fluorochrome used (e.g. CD4-FITC).

☒ The axis scales are clearly visible. Include numbers along axes only for bottom left plot of group (a 'group' is an analysis of identical markers).

☒ All plots are contour plots with outliers or pseudocolor plots.

☒ A numerical value for number of cells or percentage (with statistics) is provided.

Methodology

Sample preparation	Detailed information was provided in Methods under headings Flow cytometric analysis and DR-GFP assay.
Instrument	BD LSR II and BD FACScan
Software	Cellquest Pro v5.2, BD FACSDiva v7, Flowjo v10
Cell population abundance	Whole populations were analyzed.
Gating strategy	For cell death analysis, the cells were only gated as Annexin V positive and negative cells.<br>For DR-GFP assay, cell debris were excluded by SSC-A and FSC-H gating, doublets were excluded by FSC-A and FSC-H gating. Cells were then gated as BFP positive cells followed by GFP positive cells.

☒ Tick this box to confirm that a figure exemplifying the gating strategy is provided in the Supplementary Information.
